# Increasing wildfire frequency decreases carbon storage and leads to regeneration failure in Alaskan boreal forests

**DOI:** 10.1186/s42408-025-00390-3

**Published:** 2025-10-09

**Authors:** Xanthe J. Walker, Michelle C. Mack, Betsy Black, Jacqueline Dean, Lauren F. Kemper, Stefano Potter, Brendan M. Rogers, Charles M. Truettner

**Affiliations:** 1 Center for Ecosystem Science and Society, Department of Biological Sciences, Northern Arizona University, Flagstaff, AZ 86011 USA; 2 School of Informatics, Computing and Cyber Systems, Northern Arizona University, Flagstaff, AZ 86011 USA; 3 Pacific Southwest Research Station (ORISE), USDA Forest Service, Redding, CA 96002 USA; 4 Woodwell Climate Research Center, Falmouth, MA 02540 USA; 5 USDA American Forests, Climate Science, Washington, DC 20005 USA

**Keywords:** Alaska, Black spruce, Boreal forest, Wildfire, Carbon, Fire frequency, Fire regime, Fire return interval, Resilience, Successional trajectory

## Abstract

**Background:**

The increasing size, severity, and frequency of wildfires is one of the most rapid ways climate warming could alter the structure and function of high-latitude ecosystems. Historically, boreal forests in western North America had fire return intervals (FRI) of 70–130 years, but shortened FRIs are becoming increasingly common under extreme weather conditions. Here, we quantified pre-fire and post-fire C pools and C losses and assessed post-fire seedling regeneration in long (> 70 years), intermediate (30–70 years), and short (< 30 years) FRIs, and triple (three fires in < 70 years) burns. As boreal forests store a significant portion of the global terrestrial carbon (C) pool, understanding the impacts of shortened FRIs on these ecosystems is critical for predicting the global C balance and feedbacks to climate.

**Results:**

Using a spatially extensive dataset of 555 plots from 31 separate fires in Interior Alaska, our study demonstrates that shortened FRIs decrease the C storage capacity of boreal forests through loss of legacy C and regeneration failure. Total wildfire C emissions were similar among FRI classes, ranging from 2.5 to 3.5 kg C m^−2^. However, shortened FRIs lost proportionally more of their pre-fire C pools, resulting in substantially lower post-fire C pools than long FRIs. Shortened FRIs also resulted in the combustion of legacy C, defined as C that escaped combustion in one or more previous fires. We found that post-fire successional trajectories were impacted by FRI, with ~ 65% of short FRIs and triple burns experiencing regeneration failure.

**Conclusions:**

Our study highlights the structural and functional vulnerability of boreal forests to increasing fire frequency. Shortened FRIs and the combustion of legacy C can shift boreal ecosystems from a net C sink or neutral to a net C source to the atmosphere and increase the risk of transitions to non-forested states. These changes could have profound implications for the boreal C-climate feedback and underscore the need for adaptive management strategies that prioritize the structural and functional resilience of boreal forest ecosystems to expected increases in fire frequency.

**Supplementary Information:**

The online version contains supplementary material available at 10.1186/s42408-025-00390-3.

## Resumen

**Antecedentes:**

El incremento en tamaño, severidad, y frecuencia de los incendios de vegetación es una de las formas más rápidas en la que el calentamiento climático puede alterar la estructura y función de los ecosistemas ubicados a latitudes altas. Históricamente, los bosques boreales en el oeste de Norteamérica tuvieron intervalos de retorno de fuego (FRI) de entre 70 y 130 años, aunque la reducción del período de FRI está siendo incrementalmente común bajo condiciones climáticas extremas. En este trabajo, cuantificamos los depósitos de C pre y post fuego, las pérdidas de C, y determinamos la regeneración de plántulas post fuego en períodos largos (> 70 años), intermedios (30 -70 años), y cortos (< 30 años) de FRI, y en tres incendios en < 70 años. Dado que los bosques boreales almacenan una cantidad significativa del Carbono (C) terrestre, el entender los impactos de la reducción de las FRI en esos ecosistemas es crítico para predecir el balance global del C y sus retroalimentaciones con el clima.

**Resultados:**

Usando un conjunto de datos espacialmente extenso de 555 parcelas de 31 incendios ocurridos en el interior de Alaska, nuestro estudio demostró que la reducción en el FRI disminuye la capacidad de almacenamiento de C de los bosques boreales a través de pérdidas en el C acumulado, y en fallas en la regeneración. El total de las emisiones por los incendios fue similar entre las clases de FRI, y variaron entre 2.5 y 3.5 kg m-2. Sin embargo, el acortamiento de los períodos de FRI hizo que el bosque perdiera más C que estaba acumulado en el pre-fuego, resultando en un almacenaje sustancialmente menor que en los casos en que el FRI fuese más largo. Períodos más cortos de FRI también resultaron en una pérdida del legado del carbono almacenado, definido como el carbono que se consumió por combustión en uno o más de los incendios previos. Encontramos asimismo que las trayectorias sucesionales post fuego fueron impactadas por el FRI, con un ~ 65% de intervalos cortos de FRI e incendios triples que experimentaron fallas en la regeneración.

**Conclusiones:**

Nuestro estudio subraya la vulnerabilidad estructural y funcional de los bosques boreales al incremento de las FRI. El achicamiento de las FRI y la combustión del legado de C pueden modificar los ecosistemas boreales, de ser un almacén neto de carbono o ser neutral (sin pérdidas ni ganancias de C), a transformarse en una fuente de emisiones de C hacia la atmósfera e incrementar el riesgo de hacer una transición hacia estados no forestales. Esos cambios podrían tener profundas implicancias la retroalimentación del clima boreal y subraya la necesidad de estrategias de manejo adaptativas que prioricen la resiliencia estructural y funcional de los ecosistemas boreales al esperado incremento en la frecuencia de los incendios.

## Background

Climate-induced increases in wildfire activity could alter the structure and function of high-latitude ecosystems. The historical fire return interval (FRI) of boreal forests in Western North America ranges from 70 to 130 years (Johnstone, Chapin, et al. 2010), but short FRIs (< 30 years are becoming increasingly common under extreme weather conditions) (Whitman et al. 2024). A shortened FRI, often called reburning (Prichard et al. 2017), can shift boreal ecosystems across a carbon (C) cycle threshold: from a net accumulation of C from the atmosphere over centuries and fire cycles to a net loss, acting as a positive feedback to climate warming (Walker et al. 2019). Reburning not only has the potential to impact the net ecosystem C balance (NECB) but also sets the conditions that control tree seedling recruitment and, therefore, tree dominance (Hayes and Buma 2021; Johnstone, Hollingsworth, et al. 2010), and long-term C storage (Mack et al. 2021). Understanding the ecological consequences of increased fire frequency and a shortened FRI is critical for predicting the future state of boreal forests and the global C cycle.

Wildfire activity in the boreal forests is controlled by top-down forcings of climate and weather (Bessie and Johnson 1995; Prichard et al. 2017) and bottom-up drivers of fuel composition and quantity (Bernier et al. 2016; Parks et al. 2018; Erni et al. 2018). The most widespread example of a bottom-up control on fire activity is age-dependent burning, where young stands are unlikely to burn until fuels accumulate to levels that can sustain fire (Parks et al. 2015). In boreal forest ecosystems, recently burned areas generally resist burning for upwards of 30 years after a fire (Erni et al. 2018; Parks et al. 2018). Managers often use these recently burned areas as a natural fuel break, relying on the reduced fuel loads to limit the spread of subsequent fires (Agee and Skinner 2005; Stephens and Ruth 2005). However, climate and the associated effects of climate change on the fire regime are already beyond the historical range of variability (Kelly et al. 2013). Increases in extreme fire weather conditions can override age-dependent burning and allow reburning to occur (Parks et al. 2015; Whitman et al. 2024; Hayes et al. 2024).

Continued increases in fire frequency and the burning of young-aged stands with relatively low fuel loads will likely impact the long-term C storage capacity of boreal forest ecosystems. In mature boreal forests, the majority of C stored and lost from combustion is from the soil organic layer (SOL; Walker et al. 2020a, b). A shortened FRI limits the time for SOL reaccumulation and can shift boreal ecosystems to a net C loss when burning releases SOL legacy C, defined as C that escaped combustion in one or more previous fires (Walker et al. 2019). The loss of legacy C represents a state change from the historical net C sink or neutral to a net C source to the atmosphere over consecutive fires (Chapin et al. 2006; Walker et al. 2019). Given that these forests and cold soils store approximately 30% of global terrestrial C in a pool twice the size of the atmospheric C pool (Schuur et al. 2018), changes in C release and storage from increased fire frequency could fundamentally alter global climate (Field et al. 2007; Lenton et al. 2019).

Increased boreal wildfire activity can also indirectly impact NECB through altered successional trajectories (Mack et al. 2021). Much of the North American boreal forest is dominated by conifer stands of black spruce (*Picea mariana*). For the past ~ 6000 years, there has been a persistent cycle of black spruce self-replacement following fires Lloyd et al. 2006, Higuera et al. 2009). Deep burning of the SOL can change patterns of post-fire recruitment, favoring rapidly growing broadleaf deciduous trees such as aspen (*Populus tremuloides*) and birch (*Betula neoalaskana*; Johnstone and Chapin 2006, Baltzer et al. 2021). Rapid declines in seedbed quality effectively restrict seedling establishment to a short post-fire window, meaning that initial patterns of tree seedling recruitment are representative of future forest composition (Charron and Greene 2002; Johnstone et al. 2004, 2020). Shortened FRIs that limit SOL reaccumulation (Walker et al. 2019) and the development of an aerial seed bank (Viglas et al. 2013; Alfaro-Sánchez et al. 2022) can lead to deciduous dominance, recruitment failure, and transition to grasslands or shrublands (Pinno et al. 2013; Whitman et al. 2019; Brown and Johnstone 2012; Hayes and Buma 2021). Once deciduous forests are established via seed following wildfire, their asexual regeneration strategies facilitate rapid and prolific post-fire colonization (Walker et al. 2023). However, a shortened FRI could impede the effectiveness of these regeneration strategies due to immature trees having insufficient carbohydrate reserves for resprouting (Clarke et al. 2013).

Using a spatially extensive dataset of 555 plots from 31 separate fires in Interior Alaska, we describe the forests and landscape characteristics of reburns and ask what the consequences of changing FRIs are for C pools and successional trajectories. Specifically, we quantified pre-fire and post-fire C pools and C losses and assessed post-fire seedling regeneration in long (> 70 years), intermediate (30–70 years), and short (< 30 years) FRIs, and triple (three fires in < 70 years) burns. We then test the hypotheses that reburning will:

1. Decrease pre- and post-fire C pools and increase legacy C combustion due to the shortened time interval between fires limiting SOL reaccumulation, where most C is stored in these ecosystems.
2. Increase the frequency of alternate post-fire successional trajectories in black spruce stands due to the loss of the aerial seed bank and increased mineral soil exposure but will not impact regeneration in deciduous stands due to their ability to rapidly resprout following wildfire.

Results from this research provide insight into the structural and functional resilience of boreal forests to increasing fire frequency, which is needed to develop management strategies that promote ecosystem resilience. Furthermore, most research on fire impacts in boreal Alaska, and specifically impacts on C loss and storage, come from relatively mature black spruce forests. By examining reburned ecosystems, we provide a basis for anticipating the consequences of altered fire regimes on future forest composition and feedbacks to the global C cycle and climate.

## Methods

### Study area and site selection

**Fig. 1:**
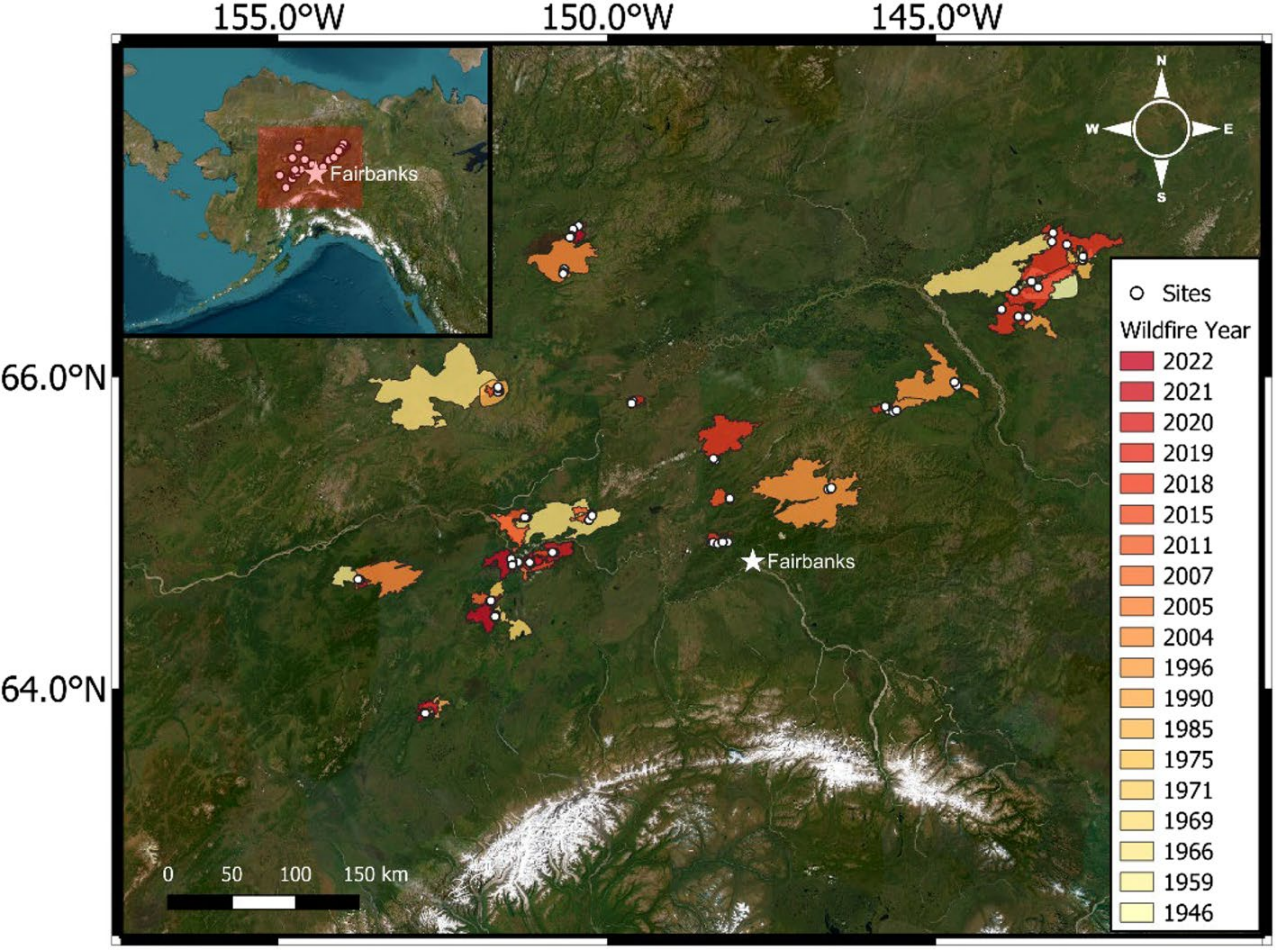
Locations of sites and fires colored by burn year

Our study took place within Interior Alaska, USA, ranging from south of the Brooks Range to north of the Alaska Range (Fig. 1). The majority (57%) of the 370,000 km^2^ Alaskan boreal forest is conifer-dominated, 27% is a mix of conifer and deciduous trees, including trembling aspen (*Populus tremuloides*) and paper birch (*Betula neoalaskana*), and 16% is treeless wetlands and tundra (Neigh et al. 2013; Yarie and Billings 2002). The majority (75–80%) of the boreal forest in Interior Alaska is underlain by permafrost (Osterkamp & Romanovsky 1999), which tends to be absent from south-facing slopes and floodplains (van Cleve et al. 1983). Large areas of Interior Alaska have burned, resulting in fires overlapping with one or more previous fires and providing an opportunity to sample a range of FRIs.

Reburned fire locations were identified along the road network and within a 150-km radius of both Fort Yukon and Manley, AK. We limited our fire selection to areas we could sample within 7 years following fires to ensure minimal C reaccumulation and accurate measures of residual C pools and C loss. The Alaska Large Fire Database (ALFD) along with 30 m Landsat-based differenced Normalized Burn Ratio (dNBR) was used to verify locations that reburned between 2015 and 2022. We selected potential field sampling locations by creating a 100-m grid of points across each reburned fires, with a 100 m buffer from the edge of the fire polygon. The dNBR at each point was extracted, and all points with a dNBR value of 0.05 or less were removed to exclude unburned points. Potential sampling locations were stratified by fire severity of the most recent fire. Severity was assigned by converting reburned dNBR into three quantiles representing low, medium, and high severity. We used dNBR distribution across all reburned fires to classify fire severity. Finally, we selected long FRI sites (i.e., sites that burned in the same fire year as reburns between 2015 and 2022 but had no recorded history of fire prior to that) in a similar manner, stratified by severity across all long FRI fires.

In July and August of 2021, 2022, and 2023, we sampled 31 fires throughout Interior Alaska. In each fire, we sampled five to nine randomly located sites, with at least two sites in each dNBR fire severity class when available (e.g., some fires only had two of the three severity classes present), for a total of 185 sites. Within each site, we established three replicate sampling plots, each measuring 10 m × 2 m, for a total of 555 plots. We acquired downscaled climate data from ClimateNA (Wang et al. 2025) to calculate the mean annual temperature, mean annual precipitation, and mean annual climate moisture index (CMI) from 1985 to 2015 for each of our sampled sites.

### Field methods

At each site, we recorded latitude, longitude, and elevation with a GPS receiver, and slope and aspect with a clinometer and compass. Each plot was assigned a soil drainage class on a six-point scale, ranging from xeric to subhygric, based on topography‐controlled drainage and adjusted for soil texture and the presence of permafrost (Johnstone, Hollingsworth and Chapin 2008).

To estimate stand age at the time of fire, we collected one basal tree cross-section in each plot from the dominant pre-fire tree species (three trees per site). If co-dominant tree species were present pre-fire, then one cross-section per tree species was collected for each plot. In each plot (20 m^2^), we measured the diameter at breast height (DBH; 1.37 m from the base) for all trees ≥ 1.37 m in height and the basal diameter of all trees < 1.37 m tall that were present pre-fire. For each tree, we assessed the percent combustion (0%, 25%, 50%, 75%, 100%) for the stem, branches, coarse branches, fine branches, foliage/needles, and cones (if applicable) from the most recent fire. We similarly measured pre-fire density, composition, and combustion of tall shrubs (i.e., *Salix*, *Alnus*, and *Betula* spp.) for each plot. We measured basal diameter and estimated percent combustion of stem, branches, and foliage for all shrubs rooted within 10 m^2^ (one-half of the belt transect). We also measured all coarse woody debris (CWD) > 2 cm in diameter that intersected the transect line (Brown 1974). All CWD that was lying on the ground or situated at a < 45° angle from the ground was included. For all CWD, we recorded species (if identifiable), diameter (cm), decay class (hard, crumbly, soft; Manies et al. (2005)), and visually estimated percent combustion.

Thaw depth and residual SOL measurements were made at two to three evenly spaced locations per plot (three points per plot in 2021 and two points per plot in 2022 and 2023) for a total of 1262 thaw and residual SOL depth measurements. Thaw depth was measured by physically probing the ground and recording the presence and depth of the underlying frozen ground or rocks. Given that thaw depth was measured mid-July through mid-August in each year, this is a metric of permafrost presence rather than peak thaw depth. Residual SOL was measured from the SOL surface to the organic mineral interface. Paired with each transect measurement, we also measured the residual SOL depth as close as possible to the base of the nearest black spruce tree (when present). For these trees, we measured the height of the adventitious roots to the top of the residual SOL on 1–3 adventitious roots per tree to estimate burn depth (see below).

We collected the entire residual SOL profile at one point per plot along the transect. An intact SOL sample of approximately 10 cm × 10 cm × variable depth was collected using a serrated knife and pruners. Dimensions of each SOL sample were recorded in the field. Samples were frozen and returned to the laboratory for processing.

To measure post-fire seedling recruitment, our indicator of shifting species dominance and successional trajectories, we established two to three 1 m × 1 m quadrats within each plot. These were paired with the SOL and thaw depth measurements for a total of 1262 quadrats across the network of plots. We counted all tree seedlings and resprouts and noted species composition. We measured the basal area of up to three representative seedlings per species per quadrat. If no seedlings or resprouts were measured within the sampled quadrats, but were visible at the site, we randomly sampled additional quadrats until we encountered an individual.

### Laboratory methods

Tree cross-sections were processed using standard dendrochronology techniques (Cook and Kairiukstis 1990), and tree rings were counted to estimate stand age at the time of fire. For sites with no recorded history of fire, we used tree ring counts to estimate mean stand age. For reburned sites, if the tree ring data corroborated the fire history data (e.g., all trees were approximately the same age as the previous fire) we used the fire history data. If fire history did not align with tree ring data, we used the tree ring data because of the likelihood of unburned patches (Dieleman et al. 2020). Based on fire history data, we sampled 53 long (> 70 years), 44 intermediate (30–70 years), 60 short (< 30 years), and 28 triple (three fires in 70 years) burned sites. Tree ring data did not align with fire history in 14% of our sites, so we reclassified five intermediate FRI sites to long, 11 short to long, five short to intermediate, two triple to long, and three long to intermediate, resulting in 68 long, 47 intermediate, 44 short FRI sites, and 26 triple burned sites. Of the triple burned sites, five experienced two subsequent intermediate FRIs, eight were a short interval followed by an intermediate, 12 were an intermediate followed by short, and one site experienced two subsequent short FRIs. We classified the triple burns into 13 triple-mid (the most recent interval 30–70 years) and 13 triple-short (the most recent interval < 30 years) burned sites.

In the laboratory, we thawed SOL monoliths at room temperature (~ 25 °C) for approximately 24 h. We bisected a subset (*n* = 319) of our collected SOL monoliths depth-wise using an electric carving knife and saved one-half of the monolith for archival purposes. On the remaining half, green moss, and vascular plants were discarded. We then divided them into 5-cm depth increments, with the deepest sample being of variable depth, depending on the location of the organic/mineral soil interface. Two measurements of depth, height, and width were obtained from each increment, and wet weight was recorded. The 5-cm increment samples from all monoliths were homogenized by hand and rocks, sticks, or coarse roots > 2 mm in diameter were removed. Fine (< 2 mm) and coarse (> 2 mm) organic fractions were weighed wet, dried at 60 °C for 48 h, and reweighed to determine dry matter content. Rock volume was estimated by water displacement in a graduated cylinder. Fine organic fractions were ground. We determined percent C using a Costech Elemental Analyzer calibrated with the NIST 1547 peach leaf standards. We determined the bulk density of fine organic fractions for each sample by dividing total dry weight by sample volume and subtracting rock volume. We calculated the C pool by multiplying the sample depth by the bulk density and percent C of the sample. In total, we sampled 634 depth wise increments from 303 soil monoliths in 151 sites. We calculated the residual soil C pool by summing the C content of all the depth-wise soil increments within a soil profile.

### Data processing and statistical analyses

All data manipulations and statistical analyses were conducted in the statistical software program R version 4.4.1 (R Core Team 2024). Data arrangements and basic calculations were done using the “dplyr” package (Wickham et al. 2018). Generalized linear mixed-effects models (GLMM) were completed using the “glmmTMB” package (Brooks et al. 2017). For all GLMM that follow, the significance of fixed effects was assessed using likelihood ratio tests of the full model against reduced models and verified using AIC, where ΔAIC > 2 denotes a difference in model performance (Zuur et al. 2009). We verified that statistical assumptions of homogeneity of variance and normality of residuals were not violated using simulated residuals from the “DHarma” package (Hartig 2020).

Stem counts, diameter measurements, and published allometric equations (Alexander et al. 2012) were used to calculate tree density (number stems m^−2^), basal area (m^2^ ha^−1^), and aboveground biomass (kg dry matter m^−2^) of the total tree, bark, main branches, fine branches, and needles/leaves, for each tree species in each plot. Total tree biomass combusted was calculated per tree from the assigned combustion class and affected biomass components (foliage, branches, and bark). We summed individual tree estimates and divided them by the sample area to estimate pre-fire biomass and biomass combustion (kg dry matter m^−2^) per plot. We similarly used published allometric equations (Berner et al. 2015) to calculate biomass and combusted biomass for each shrub species. For CWD, we used the size classes from Nalder et al. (1997), decay classes from Manies et al. (2005), and biomass calculations from Ter Mikaelin et al. (2008) to estimate the pre-fire, post-fire, and combusted biomass pools from CWD. To calculate the pre-fire, post-fire, and combusted C pools of trees, shrubs, and CWD, we assumed a biomass C content of 50%.

Following Mack et al. 2021 and Walker et al. 2023, we classified plots into pre-fire tree species dominance classes based on a deciduous fraction index (DI) calculated from the survey data (DI = relative density plus relative biomass of deciduous trees divided by two and multiplied by 100). When DI was ≤ 33.33%, plots were classified as spruce. Plots were classified as mixed spruce-deciduous (hereafter mixed) if DI was > 33.33% and < 66.66%, and as deciduous if DI was ≥ 66.66%. Plots were classified as open if no trees were measured within the site. To determine the relationship between FRI and pre-fire composition class, we completed a chi-square test of independence with simulated *p*-values based on 10,000 replicates. We used a post hoc test in the “chisq.posthoc.test” package (Ebbert and Ebbert 2019) with a false discovery rate adjustment to test which pre-fire composition occurred more or less frequently than expected in each FRI.

To calculate the SOL burn depth in plots where black spruce trees and adventitious roots were present pre-fire, we used the adventitious root method and calibrations described in Boby et al. (2010), in which burn depth is equivalent to the height of adventitious roots (ARH) above the residual SOL plus an offset of 3.2 cm associated with the depth of AR in the SOL. The total depth of pre-fire SOL was then reconstructed by adding the burn depth to the residual SOL depth. We completed this for 832 ARH measurements from 357 plots nested within 125 sites and 26 fires.

For plots where no adventitious roots were present (i.e., Young spruce, *n* = 57 plots), Deciduous (*n* = 100 plots), Mixed (*n* = 26 plots), or Open (*n* = 14 plots), we estimated pre-fire SOL depth using random forest models trained with data acquired from forest inventory databases (Supplementary Material 2). We estimated burn depth as the difference between pre-fire SOL depth based on modeled estimates and measured residual SOL depth. We completed these calculations for 428 residual SOL measurements from 197 plots nested within 71 sites and 19 fires. If the residual SOL measurement was greater than the modeled estimated pre-fire SOL depth, burn depth was assigned a value of zero, and pre-fire SOL depth was assumed to be the same as residual SOL depth.

We predicted residual soil C pools for every SOL depth measurement (*n* = 1262) using both our collected SOL samples and post-fire SOL C pools from additional datasets that were sampled by our research group following fires in Interior Alaska (Boby et al. 2010; Walker et al. 2021, Black et al. in review). In total, we used soil C data from 1547 depth-wise soil increments from 641 soil monoliths located in 293 distinct sites in Interior Alaska, including the 688 depth-wise increments from 319 soil monoliths collected as part of this study. Following the methods outlined in Walker et al. (2020a, b), we modeled residual SOL C pools (g C m^−2^) as a function of depth and depth class (0–10 cm, 10–20 cm, and > 20 cm) and their interaction using a GLMM. We assigned each site a forest composition class and fit a separate GLMM for each class of Spruce (*n* = 925), Deciduous (*n* = 93), Mixed (*n* = 112), and Open (*n* = 315). All models included a random intercept of site. We used a gamma distribution with a “log” link to ensure our models met the distributional assumptions. We used these models to predict residual SOL C pools (g C m^−2^) based on the residual SOL depth measurements (total = 1262) for each pre-fire class of Spruce (*n* = 850), Deciduous (*n* = 253), Mixed (*n* = 129), and Open (*n* = 30). For our final estimate of residual SOL C pools, we combined the measured data (*n* = 319) with the modeled estimates (*n* = 943) and calculated plot-level averages.

We similarly predicted soil C combusted (g C m^−2^) for every estimate of burn depth (*n* = 1262) using datasets from 111 distinct sites in Interior Alaska that had no recorded history of fire (Alexander et al. 2012; Boby et al. 2010; Melvin et al. 2015; Walker et al. 2021). These datasets included 1346 depth-wise soil increments from 541 soil profiles. We created separate models for soil C using the same GLMM structure described above for each of the four forest composition classes of Spruce (*n* = 1027), Deciduous (*n* = 164), Mixed (*n* = 81), and Open (*n* = 74). We applied these depth-wise C pool models to burn depth estimates for each pre-fire class of Spruce (*n* = 850), Deciduous (*n* = 253), Mixed (*n* = 129), and Open (*n* = 30) to predict soil C combusted (g C m^−2^) and then calculated plot-level averages.

To estimate pre-fire SOL C pools (g C m^−2^), we summed either the measured or modeled residual C pools with the modeled combusted C pools, which were then averaged per plot. Using the plot-level estimates of aboveground and belowground C pools, we calculated total pre-fire, post-fire, and combusted C pools. To determine how C pools differ among FRIs, we fit GLMMs for aboveground, belowground, and total pre-fire, post-fire, and combusted C pools. We also fit GLMMs for pre-fire, post-fire, and combusted C pools for each aboveground component (CWD, snags, shrubs, and trees). We used a Tweedie distribution for each model with site as a random intercept and FRI (5 levels) as a fixed effect. We used a post hoc analysis for multiple comparisons in the R package “emmeans” (Lenth et al. 2019) with a false discovery rate adjustment to test for differences in marginal means among FRI classes.

We estimated the amount of both belowground and aboveground legacy C combusted—C that escaped combustion in one or more previous fires—for each of the reburned FRI classes. Belowground legacy C is the SOL C that escaped combustion, whereas aboveground legacy C is the CWD and deadwood that remains following fire. For both belowground and aboveground pools, legacy C combustion was calculated as the difference between the C pool accumulated during the fire-free interval and C lost in the most recent fire. Carbon accumulation for the mid and short FRIs was estimated as the difference between the pre-fire C pool of the class and the mean post-fire C pools of long FRIs. This was because all mid and short classes experienced a long FRI prior to the recent burn. Carbon accumulation in the triple classes was estimated as the difference between the pre-fire C pool of the class and the mean post-fire C pool of the mid-FRIs. This is because most of the triple burns (17 out of 26) experienced a mid-FRI prior to the recent burn. Using this method, we could not calculate legacy C loss for long FRIs as the net accumulation is equivalent to the C combustion. The percent of C combustion attributed to legacy C was calculated as legacy C loss divided by total C loss.

We calculated the average density and biomass of each tree seedling in each site using species-specific seedling counts, basal diameter measurements, and allometric equations (Johnstone et al. 2020). We summed all sample quadrats in each site to ensure sufficient sample area (6 to 9 m^2^) to capture the spatial heterogeneity in seedling recruitment. As post-fire seedlings might not be present in the year immediately following a fire, we only included sites that were measured two or more years after fire (134 out of 185 sites). This resulted in 54 long FRIs, 33 mid, 32 short, 8 triple-mid, and 7 triple-short. We then calculated the DI previously described for post-fire seedlings and classified sites into post-fire tree species dominance classes. All sites with no seedlings present were classified as open (i.e., regeneration failure). This cut-off is intentionally conservative, such that sites with low regeneration density (< 1 seedling per m^2^) are not classified as open. This approach ensures a robust classification of post-fire regeneration that accounts for sparse recruitment and within-site variability.

To determine the relationship between FRI and post-fire successional trajectories for all sites, we completed chi-square tests of independence with simulated *p*-values based on 10,000 replicates. We followed this with a post hoc test in the package “chisq.posthoc.test” (Ebbert and Ebbert 2019) with a false discovery rate adjustment to test which successional trajectories occurred more or less frequently than expected. We used the same tests to explore the resilience of pre-fire spruce-dominated sites (*n* = 83) across FRIs (46 long FRIs, 28 mid, 6 short, 1 triple-mid, and 3 triple short). There were too few pre-fire deciduous sites (*n* = 31), mixed (*n* = 15), and open (*n* = 4) sites across FRIs to accurately test the independence of successional trajectories among FRIs within these pre-fire composition classes.

For all sites (*n* = 134) and for the pre-fire black spruce-dominated sites (*n* = 83), we modeled how post-fire spruce and deciduous density varied among FRIs. For each model, we fit a GLM with a Tweedie distribution and FRI as a fixed effect. For models using all sites, we excluded the triple-mid FRI as deciduous and spruce density was always zero. For the pre-fire spruce models, we had to exclude the triple-mid FRIs for the post-fire conifer density model and both the triple-mid and triple-short for the post-fire deciduous model. We used a post hoc analysis for multiple comparisons in the R package “emmeans” (Lenth et al. 2019) with a false discovery rate adjustment to test for differences in marginal means among FRI classes.

## Results

### Characterizing reburns

**Fig. 2:**
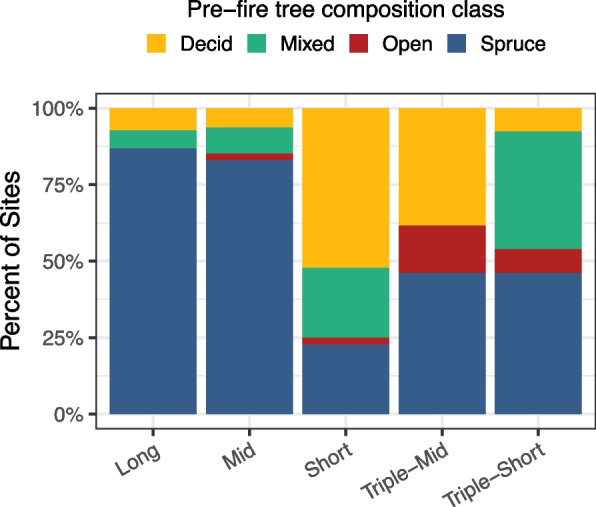
Percent of sites distributed among different pre-fire tree species composition classes for each fire return interval (long > 70 years, mid > 30–70 years, short < 30 years, triple-mid: three fires in < 70 years, with the most recent interval of 30–70 years, and triple-short: three fires in < 70 years, with the most recent interval of < 30 years). Colors represent pre-fire tree species composition based on a deciduous fraction index (DI = relative density plus relative biomass of deciduous trees divided by two and multiplied by 100). When DI was ≤ 33.33%, plots were classified as Spruce (blue). Plots were classified as Mixed (green) if DI was > 33.33% and < 66.66%, and as Deciduous (yellow) if DI was ≥ 66.66%. Plots were classified as Open (red) if there were no trees measured within the site. See Table S1 for results of chi-square test of independence between FRI and pre-fire tree species composition classes

We examined 555 burned-forest plots nested within 185 sites that captured a broad gradient in environmental characteristics, pre-fire stand composition, and FRI (Table 1, Fig. 2). Within each FRI category, we captured the full range of moisture conditions from xeric to subhygric. However, short FRIs were generally located in drier and long FRIs in wetter landscapes positions (Table 1). The frequency of sites where we detected permafrost was substantially higher in long FRIs (47% of sites) than in intermediate (26%) or short (14%) FRIs. Interestingly, we detected permafrost at 39% of the triple-mid sites, but only 15% of the triple-short. The frequency of rocky soils were similar among long, intermediate, and short FRIs, but were not encountered in the triple-burned sites. Our extensive sampling indicates that reburns occur across a range of landscapes but are generally in drier permafrost-free areas compared to long FRI sites.

**Table 1:** Climate, landscape, and pre-fire characteristics of sites examined in this study across fire return intervals. Values represent mean ± standard error of the mean and the range

	**Variable**	**Long**	**Intermediate**	**Short**	**Triple-Mid**	**Triple-Short**
	# of sites (plots)	68 (204)	47 (141)	44 (132)	13 (39)	13 (39)
Climate	MAT (C)	− 4.4 ± 0.07	− 3.7 ± 0.09	− 4.5 ± 0.08	− 4.5 ± 0.18	− 3.8 ± 0.16
	MAP (cm)	17.43 ± 0.23	17.48 ± 0.26	17.28 ± 0.27	17.36 ± 0.45	16.92 ± 0.70
	CMI (cm)	0.39 ± 0.01	0.41 ± 0.01	0.29 ± 0.01	0.23 ± 0.01	0.42 ± 0.02
Landscape	Elevation (m.a.s.l.)	354.18 ± 24.32 (77–834)	302.30 ± 25.06 (77–852)	357.48 ± 26.74 (76–676)	318.62 ± 43.21 (128–531)	235.15 ± 30.89 (138–517)
	Slope (radians)	0.10 ± 0.01 (0–0.37)	0.07 ± 0.01 (0–0.56)	0.12 ± 0.02 (0–0.59)	0.07 ± 0.03 (0.02–0.35)	0.03 ± 0.01 (0–0.12)
	Aspect (radians)	2.87 ± 0.27 (0–6.25)	2.23 ± 0.30 (0–6.02)	2.60 ± 0.31 (0–5.93)	3.09 ± 0.45 (1.05–6.13)	1.44 ± 0.58 (0–6.11)
	Heatload	0.69 ± 0.03 (0.19–1.15)	0.71 ± 0.04 (0.23–1.14)	0.69 ± 0.04 (0.20–1.15)	0.65 ± 0.11 (0.22–1.11)	0.54 ± 0.07 (0.20–0.94)
	Moisture (unitless)	4.09 ± 0.18 (1–6)	3.36 ± 0.14 (1–6)	2.61 ± 0.18 (1–5)	3.38 ± 0.27 (2–6)	3.54 ± 0.49 (1–6)
	% of sites with permafrost	47.10%	25.50%	13.60%	38.50%	15.40%
	Frequency of rocks	0.18 ± 0.04 (0–1)	0.19 ± 0.05 (0–1)	0.24 ± 0.06 (0–1)	0 ± 0 (0–0)	0 ± 0 (0–0)
Pre-fire	Stand age (years)	102.56 ± 3.58 (71–254)	45.73 ± 1.43 (32–70)	15.90 ± 0.54 (11–24)	39.45 ± 2.51 (32–62)	21 ± 0.97 (17–26)
	SOL depth (cm)	27.17 ± 1.37 (7.12–71.74)	19.78 ± 1.38 (6.58–43.03)	12.80 ± 1.15 (4.85–35.08)	19.13 ± 3.24 (7.61–41.41)	14.60 ± 2.50 (6.03–39.08)
	Tree density (stems m^−2^)	0.97 ± 0.10 (0.10–3.73)	1.24 ± 0.23 (0–9.40)	0.55 ± 0.15 (0–6.15)	0.62 ± 0.24 (0–2.58)	1.44 ± 0.46 (0–5.68)
	Tree basal area (cm^2^ m^−2^)	3417.78 ± 479.09 (180.56–26,108.56)	2985.15 ± 885.79 (0–41387.69)	1101.89 ± 374.89 (0–15618.54)	827.33 ± 423.40 (0–5679.04)	618.29 ± 225.90 (0–2957.87)
	Proportion black spruce (0–1)	0.88 ± 0.03 (0–1)	0.88 ± 0.03 (0.03–1)	0.36 ± 0.06 (0–1)	0.59 ± 0.14 (0–1)	0.78 ± 0.09 (0–1)

We found that FRI and pre-fire tree species dominance were related (Χ^*2*^ = 83.01, *p*-value < 0.001). In long FRIs, there were significantly more spruce-dominated sites (59 of 68) and fewer deciduous-dominated sites (Table S1, Fig. 2). Mid-FRIs were also primarily composed of spruce forests (39 of 41). In contrast, short FRIs had significantly more deciduous (23 of 44) and less spruce (10 of 44). Many of the triple mid and triple short stands were spruce dominated (6 of 13 sites each), but triple-mid and triple short had marginally more pre-fire open (2 of 13) and mixed (5 of 13) sites, respectively (Fig. 2, Table S1). We found that reburning occurs across a range of pre-fire compositions, from spruce to deciduous-dominated stands or even open shrub or grass-dominated areas.

### Carbon pools

**Fig. 3:**
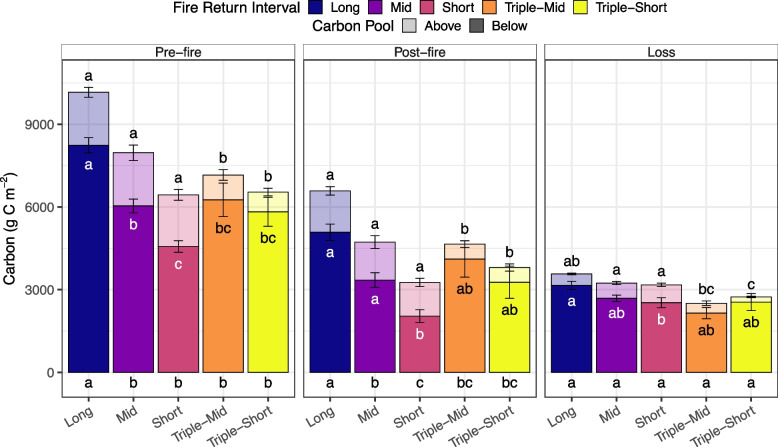
Pre-fire, post-fire, and fire losses of above- and belowground carbon (C) pools across fire return intervals (FRI; long > 70 years, mid > 30–70 years, short < 30 years, triple-mid: three fires in < 70 years, with the most recent interval of 30–70 years, and triple-short: three fires in < 70 years, with the most recent interval of < 30 years). Bars represent averaged pools for the below (dark) and aboveground (light) C pools, and error bars are the standard error of the mean. Letters represent significant differences (*P* < 0.05) among FRIs within the aboveground, belowground, and total (bottom) C pools based on generalized linear mixed models, with site as a random intercept and post hoc tests with a false discovery rate adjustment for multiple comparisons. See Table S2 for model estimated means

Pre-fire and post-fire total C pools varied among FRIs (Fig. 3, Table S2). Pre- and post-fire, short and triple-short FRIs stored ~ 35% less C, and mid and triple-mid stored ~ 25% less C than long FRIs (Fig. 3, Table S2). Total C loss was similar among FRIs, ranging from 2.5 to 3.5 kg C m^−2^. Differences in pre-fire C pools but similar C losses resulted in the proportion of pre-fire C loss being highest with short FRIs (~ 55%) compared to only 38% with long FRIs (Figure S1).

In all FRIs, most pre-fire, post-fire, and combusted C was from belowground C pools (Figure S2). Thus, the results of belowground C pools largely follow those of total C pools, with the greatest pre- and post-fire pools in long FRIs and the lowest in short FRIs (Fig. 3). Compared to long FRIs, belowground C loss was ~ 15 to 32% lower in the other FRIs. Although belowground C dominated total C pools across all FRIs, the proportion of C in belowground pools was consistently lowest in the short FRIs compared to the other FRIs; in short FRIs, 73% of pre-fire, 49% of post-fire, and 69% of combusted C were from the belowground component (Figure S2).

**Fig. 4:**
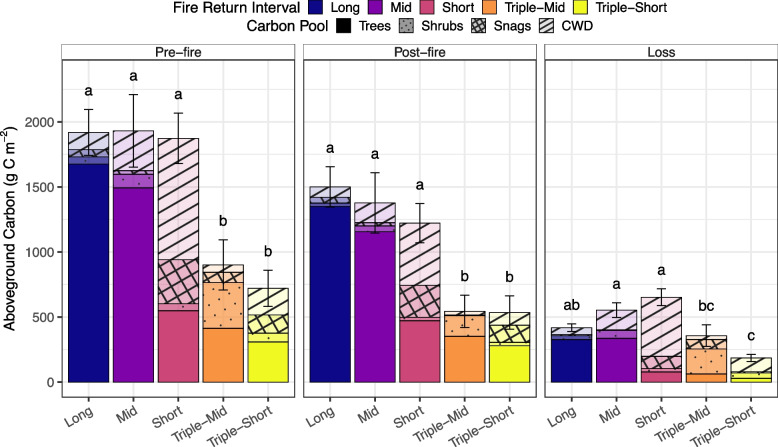
Pre-fire, post-fire, and fire losses of different aboveground carbon (C) pools across fire return intervals (FRI; long > 70 years, mid > 30–70 years, short < 30 years, triple-mid: three fires in < 70 years, with the most recent interval of 30–70 years, and triple-short: three fires in < 70 years, with the most recent interval of < 30 years). Bars represent averaged pools for each aboveground C pool (trees, shrubs, snags, and coarse woody debris), and error bars are the standard error of the mean for total aboveground C pool. Letters represent significant differences (*P* < 0.05) among FRIs for total aboveground C pools, based on generalized linear mixed models, with site as a random intercept and post hoc tests with a false discovery rate adjustment for multiple comparisons. See Table S2 for model estimated means of total aboveground C pools. See Table S3 for model estimated means and statistical differences among FRIs for each aboveground component

Aboveground pre-fire and post-fire C pools varied among FRIs, with both triple burn categories having ~ 60% lower pre-fire and post-fire pool sizes than the other FRIs (Figs. 3 and 4, and Table S1). Despite similarities in aboveground C pools among the short, mid, and long FRIs, the source of this C differed (Fig. 4 and Table S3). In long and mid FRIs, trees contributed ~ 85% of the pre-fire and post-fire aboveground C pools. In contrast, short FRIs contained over 60% of pre- and post-fire aboveground C pools in snags and CWD (Fig. 4 and Table S3). In triple burns, trees were still the primary component of aboveground C pools, but the pool was an order of magnitude smaller than those found in long and mid FRIs. Carbon loss followed these trends; in short FRIs, almost 85% of C loss was from snags and CWD, whereas in long and mid FRIs, 60–80% was from trees. In triple-mid burns, 60% of aboveground C was lost from shrubs, whereas in triple-short burns, 60% of aboveground C loss was from snags and CWD (Fig. 4 and Table S3). Ultimately, shortened FRIs shift the composition of aboveground C from tree-dominated in longer FRIs to snag, debris, or shrub-dominated systems in reburned areas.

Increased fire frequency resulted in more C lost during the most recent fire than was able to accumulate in the fire-free interval; SOL and aboveground legacy C were combusted (Table 2). This occurred with all shortened FRIs apart from the triple-mid burns. All shortened FRIs accumulated C in the fire-free interval, except for short FRIs that had lower pre-fire belowground C pools and triple burns that had lower pre-fire aboveground C pools than the estimated residual pools. Notably, ~ 40% of the aboveground C lost during short FRIs was legacy C. This percent increased by an order of magnitude in triple burns, reflecting the substantially lower pre-fire aboveground C pools in triple burns compared to the legacy C pools observed after fire. Belowground SOL legacy C also combusted in all FRIs apart from triple mid. In short FRIs, C loss was entirely from SOL legacy C pools. The percent of total C loss attributed to legacy C varied among FRIs and ranged from almost no legacy C lost with triple-mid burns to 100% with short FRIs (Table 2).

**Table 2:** Estimates of belowground, aboveground, and total legacy C combusted—C that escaped combustion in one or more previous fires—for each of the reburned FRI classes. Legacy C combustion was calculated as the difference between the C pool accumulated during the fire-free interval and C lost in the most recent fire. Carbon accumulation for the mid and short FRIs was estimated as the difference between their pre-fire C pools and the mean post-fire C pools of long FRIs. For triple burns, we used the post-fire C pools of the mid FRIs. Note that legacy C combustion cannot be estimated for the long FRIs. The amount of legacy C lost or accumulated is the difference between the accumulated C pool and C loss. The percent of C combustion attributed to legacy C (% Legacy C loss) is equivalent to legacy C lost divided by total C loss multiplied by 100

		Fire Return Interval				
		Long	Mid	Short	Triple-Mid	Triple-Short
Belowground	Postfire Residual C pool	5083	5083	5083	3350	3350
	Prefire C pool	8240	6039	4566	6260	5822
	Net C pool change in the fire free interval	3157	956	− 517	2910	2472
	Annual rate of accumulation	30.95	21.24	− 32.31	74.62	117.71
	C loss in most recent fire	3157	2689	2525	2149	2553
	Legacy C lost (neg) in most recent fire	0	− 1733	− 3042	761	− 81
	% Legacy C loss	0.00	64.45	120.48	− 35.41	3.17
Aboveground	Postfire Residual C pool	1501	1501	1501	1377	1377
	Pre-fire C pool	1919	1931	1873	901	720
	Net C pool change in the fire free interval	418	430	372	− 476	− 657
	Annual rate of accumulation	4.10	9.56	23.25	− 12.21	− 31.29
	C loss in most recent fire	418	554	652	357	186
	Legacy C lost (neg) in most recent fire	0	− 124	− 280	− 833	− 843
	% Legacy C loss	0.00	22.38	42.94	233.33	453.23
Total	Postfire Residual C pool	6584	6584	6584	4727	4727
	Pre-fire C pool	10,159	7970	6440	7161	6543
	Net C pool change in the fire free interval	3575	1386	− 144	2434	1816
	Annual rate of accumulation	35.05	30.80	− 9.00	62.41	86.48
	C loss in most recent fire	3575	3243	3177	2506	2739
	Legacy C lost (neg) in most recent fire	0	− 1857	− 3321	− 72	− 923
	% Legacy C loss	0.00	57.26	104.53	2.87	33.70

### Successional trajectories

**Fig. 5:**
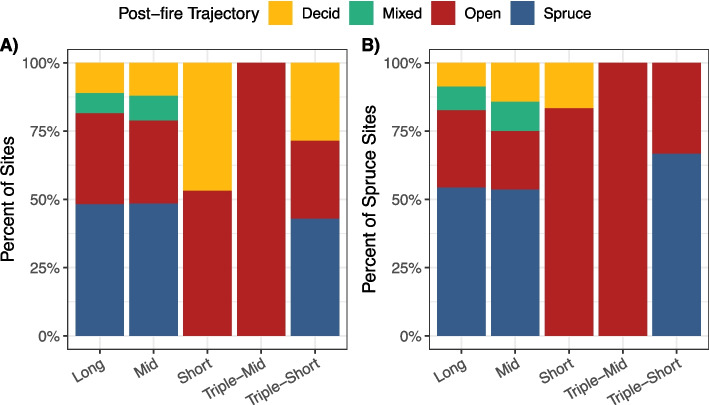
Percent of **A)** all sites and **B)** black spruce sites in each fire return interval (FRI; long > 70 years, mid > 30–70 years, short < 30 years, triple-mid: three fires in < 70 years, with the most recent interval of 30–70 years, and triple-short: three fires in < 70 years, with the most recent interval of < 30 years) that exhibited post-fire regeneration failure (red), deciduous dominance (yellow), mixed spruce-deciduous dominance (green), or spruce dominance (blue). See Table S2 for the results of chi-square tests of independence between FRI and post-fire tree species composition classes

Across all sites, we found that FRI and post-fire successional trajectory were related (*X*^2^ = 49.43, *p*-value < 0.001). Spruce the dominant post-fire trajectory for long (26 of 45 sites) and mid FRIs (16 of 33). Short FRIs were either deciduous dominated or experienced recruitment failure post-fire (Fig. 5 and Table S4). All triple-mid FRIs experienced recruitment failure, whereas 3 of the 7 triple-short sites were spruce dominant. In contrast to all sites, FRIs and post-fire successional trajectories were unrelated for pre-fire black spruce sites (*X*^2^ = 13.87, *p*-value = 0.32). Although low sample size precluded testing the relatedness of FRI and successional trajectories in pre-fire mixed, deciduous, and open stands, we observed that all pre-fire open stands remained open and ~ 60% of pre-fire deciduous and 40% of pre-fire mixed stands became open (Figure S3).

**Fig. 6:**
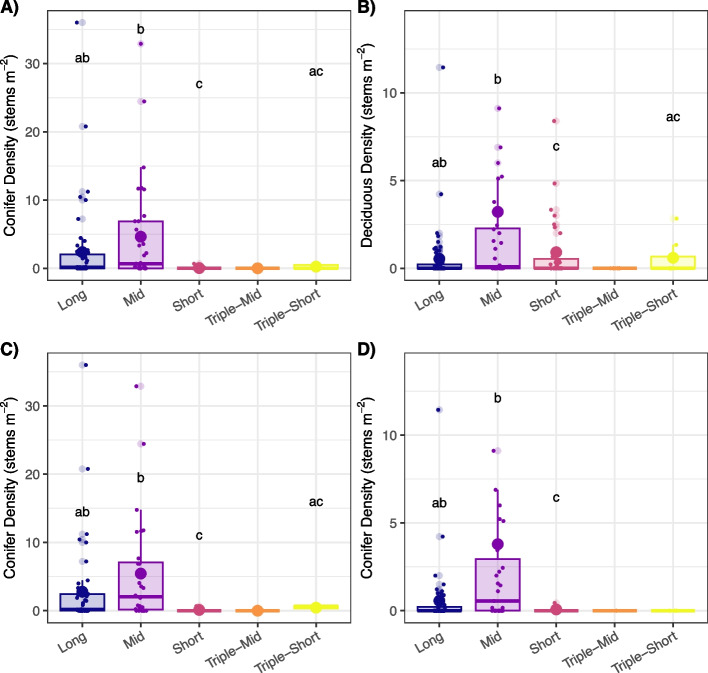
Post-fire conifer (**A**,**C**) and deciduous (**B**,**D**) seedling density in each in each fire return interval (FRI; long > 70 years, mid > 30&70 years, short < 30 years, triple-mid: three fires in < 70 years, with the most recent interval of 30–70 years, and triple-short: three fires in < 70 years, with the most recent interval of < 30 years), for all sites (**A**, **B**), and for pre-fire spruce dominated sites (**C**, **D**). Larger points represent the model estimated mean. Boxplots and more minor points are based on raw data. Letters represent significant differences (*P* < 0.05) among FRIs based on generalized linear models and post hoc tests with a false discovery rate adjustment for multiple comparisons. See Table S5 for model-estimated means

In examining post-fire density of spruce and deciduous seedlings, we saw notable differences among FRIs (Fig. 6 and Table S5). Black spruce seedling density was highest in long and mid FRIs and was < 1 seedling m^−2^ in the other FRIs. Deciduous seedling density was highest in mid-FRIs, with both long and short FRIs having similar densities (~ 0.5–0.9 seedling m^−2^). There were no deciduous or spruce seedlings in triple-mid burns and very few seedlings in triple-short burns. These results were consistent among all sites and in the subset of pre-fire black spruce sites, except for deciduous seedling density in short FRIs. Deciduous density was higher for all sites (0.9 seedlings m^−2^) compared to pre-fire black spruce sites (0.1 seedlings m^−2^; Fig. 6 and Table S5).

## Discussion

Our study demonstrates that increasing fire frequency and reburning will significantly reduce C storage capacity through loss of legacy C and regeneration failure. We examined 555 plots nested within 185 sites randomly located in 31 separate fires in boreal forests of Interior Alaska. Landscapes that experienced reburning included all forest types and topographic positions, from spruce-dominated lowlands to deciduous-dominated uplands. Our findings provide crucial conceptual understanding and calibration data for forecasting the effects of increased fire frequency on boreal ecosystems and their capacity to function as global C sinks.

To our knowledge, this is the first study to examine C combustion across a broad range of FRIs, spanning numerous fires and diverse boreal forest landscapes. Our estimates of C loss, ranging from 2.5 to 3.6 kg C m⁻^2^, align with previous results across Northwestern North American boreal forests, where total C loss ranged from 2.9 to 3.5 kg C m⁻^2^ across ecoregions and averaged 3.1 kg C m⁻^2^ in mature black spruce forests of Alaska (Walker et al. 2020a, b). We found that total C loss from wildfire was similar among FRIs but that the additive C loss of over two or three fires results in a 30–60% reduction in post-fire C stores. These findings are consistent with a case study in black spruce forests of Northwestern Canada, with a short-interval burn storing 40% less C post-fire than the long-interval burn (Brown and Johnstone 2011).

Our assessment of aboveground C pools across FRIs highlights how reburning limits the time for tree growth and biomass recovery in the fire-free interval, resulting in the combustion of deadwood (snags, CWD) and the reduction of longer-term aboveground C storage. Long and mid FRIs were characterized by live tree-dominated stands, whereas short FRIs had more deadwood, most of which was spruce. Triple burns had substantially lower aboveground C pools, with relatively little biomass in the form of deadwood compared to short FRIs. Following fire, black spruce snags typically fall and transition to CWD in 20–30 years (Bond-Lamberty et al. 2002; Boulanger and Sirois 2006). While the rate of CWD decomposition depends on substrate quality and environmental conditions, studies in Northern Quebec have shown that black spruce CWD takes 56–145 years to decompose post-falling (Boulanger and Sirois 2006). If CWD is rapidly overgrown by moss, this decay rate can be greatly reduced, resulting in buried wood that is preserved for centuries within the SOL (Moroni et al. 2010; Hagemann et al. 2010). Our study highlights that increased fire frequency leads to the instantaneous loss of legacy aboveground C in the form of deadwood that would typically slowly decompose in boreal forests.

The reduction in pre-fire SOL depth and belowground C pools with shortened FRIs relative to long FRIs emphasizes that frequent fires limit the reaccumulation of SOL and belowground C between disturbances (Walker et al. 2019). Across all FRI classes, the majority of C combusted originated from belowground in the SOL and short FRIs exhibited the highest proportional belowground C loss (~ 52% of pre-fire C) and the smallest post-fire belowground C pools. Despite experiencing two fires in < 70 years, we were surprised that triple-mid burns had slightly larger pre- and post-fire C pools than short FRIs. This is likely due to the most recent FRI being intermediate, which allows more time for SOL reaccumulation but might also be a function of triple-mid burns occurring in more poorly drained landscapes with a higher frequency of permafrost and black spruce dominance. Although boreal forests generally resist burning for upwards of 30 years, until fuels reaccumulate to levels that can sustain fire (Parks et al. 2015; Erni et al. 2018), we found that belowground C pools were reduced in all shortened FRIs compared to long FRIs. This suggests that a recovery period of < 70 years is not sufficient to reaccumulate belowground C stores between fires and highlights the potential loss of legacy C.

Similar to results in black spruce forests of the NWT, Canada, we found that shortened FRIs resulted in belowground legacy C combustion (Walker et al. 2019), with ~ 65% and 100% of the belowground C loss in mid and short FRIs, respectively, originating from C that accumulated before the most recent fire. Post-fire SOL C pools are likely to vary with fire severity, landscape position, pre-fire tree species composition, and long-term climate, and the rate of C accumulation depends on environmental conditions that impact net primary productivity and decomposition (Luyssaert et al. 2008; Jonsson and Wardle 2010; Walker et al. 2018). Thus, the lack of SOL legacy C loss in triple-mid FRIs likely represents differences in environmental conditions and the estimates of legacy SOL C combustion that we present should be considered a preliminary metric. However, even if we use the lower 95% confidence interval of post-fire belowground C pools for long FRIs, legacy SOL C combustion still accounts for ~ 100% of C loss with short FRIs and ~ 50% with mid FRIs. Any combustion of legacy C represents a change in the NECB from net C sink or neutral to a net C source and our findings highlight the vulnerability of SOL legacy C to continued increases in fire frequency.

In agreement with previous work examining post-fire recruitment with reburning (e.g., Brown and Johnstone 2012; Hayes and Buma 2021; Johnstone and Chapin 2006; Whitman et al. 2019), we observed a reduction in spruce dominance associated with shortened FRIs. In black spruce forests, increased fire frequency reduces post-fire black spruce seed availability and exposes mineral soil, leading to recruitment failure or the establishment of deciduous species (Johnstone and Chapin 2006, Brown and Johnstone 2012). Notably, both long and intermediate FRIs showed regeneration trajectories dominated by black spruce. In contrast, short FRIs and triple burns erode the compositional resilience of black spruce forests and alter post-fire successional trajectories.

Recruitment failure was unexpectedly high across all stand types. Even in long and mid FRIs, 21–28% of pre-fire black spruce sites experienced recruitment failure, slightly more than the 10–25% reported in a synthesis of black spruce stands across North America (Baltzer et al. 2021). In short and triple FRI sites, recruitment failure was even more pronounced, with 7 out of 10 pre-fire black spruce sites experiencing recruitment failure. The high failure rate with short FRIs and triple burns likely results from reproductive immaturity, as black spruce stands do not produce sufficient seeds for self-replacement until ~ 50 years (Viglas et al. 2013). Surprisingly, pre-fire mixed and deciduous sites also experienced regeneration failure (Figure S3). Deciduous forests typically rely on asexual regeneration strategies, such as resprouting, to persist through subsequent fires of varying severity (Walker et al. 2023; Jean et al. 2020). However, high-severity burning has been shown to consume the lateral roots of trembling aspen and prevent the species from establishing (Wang 2003). Additionally, short FRIs might limit the time for deciduous species to accumulate carbohydrate reserves necessary for successful asexual regeneration post-fire (Clarke et al. 2013).

In addition to our findings that increasing wildfire frequency decreases C storage and leads to regeneration failure, our extensive sampling across Interior Alaska revealed several distinct attributes of reburning. We observed that reburning occurs across all forest types and landscape positions; however, short FRIs were more prevalent at drier upland positions and triple burns at wetter lowland positions. Although reburning is thought to primarily occur in coniferous forests (Buma et al. 2022) and deciduous dominance typically reduces ignitions (Krawchuk et al. 2006), fire probability (Bernier et al. 2016; Cumming 2001), and burned area (Parisien et al. 2011), both our field observations and recent modeling work (Hayes et al. 2024) indicate that reburning can occur in deciduous and mixed stands. We also found that about 10% of our sites originally classified as reburned had to be reclassified to longer FRIs, suggesting that previous fires had unburned patches or ground fires that allowed trees to survive. This survivorship indicates that increased fire frequency might shift stands from even-aged to a more heterogeneous age structure. Furthermore, it highlights that historical fire perimeter data does not capture within-perimeter variability in fire severity and tree survivorship. Our observation that reburning occurs in areas of deciduous and mixed species dominance, with previous fires including unburned patches, highlights unique attributes of reburns that should be considered in future studies on the drivers and impacts of increasing fire frequency in boreal forest ecosystems.

### Conclusions and future research

Our study highlights that reburning occurs across diverse boreal forest landscapes and that stands thought to be resistant to fire, like young, low-density deciduous-dominated stands, experienced reburning. Examining reburning over larger spatial and temporal scales could clarify the weather conditions under which different forest types are vulnerable to reburning and assess the efficacy of recently burned areas to limit fire spread. As the climate continues to warm and fire activity increases, this information is imperative for effective and adaptive wildfire management.

We found that shortened FRIs erode both above- and belowground legacy C stores. However, fire severity and rates of post-fire C accumulation can vary substantially with stand characteristics and environmental conditions. For example, Walker et al. (2019) found that belowground legacy C never accumulates in dry stands because of complete combustion, whereas it readily accumulates in wet stands because the deep SOL burns at low severity. Comparison of stand age at the time of fire with radiocarbon dating of the residual SOL C, as demonstrated by Walker et al. (2019) and Mack et al. (2011), would refine estimates of belowground legacy C combustion across stand types and environmental conditions. Although there are likely variations in legacy C combustion that we did not capture, the loss of any legacy C represents a critical tipping point for the C balance of boreal forests, which could fundamentally alter climate dynamics (Field et al. 2007; Lenton et al. 2019).

Changes in successional trajectories are also likely to alter the boreal C-climate feedback. A switch from black spruce to deciduous dominance can act as a negative or neutral feedback to climate warming via the rapid accumulation of aboveground C pools (Mack et al. 2021). However, we observed recruitment failure more frequently than transitions to deciduous dominance. The switch from forests to shrublands or grasslands has widespread implications, including reduced C storage and the potential to accelerate the boreal C-climate feedback (Burrell et al. 2021). While fire severity and frequency are critical determinants of post-fire recruitment, other factors such as post-fire climate conditions, nutrient availability, soil microbial processes, and herbivory may also play significant roles (Whitman et al. 2019; Stevens-Rumann et al. 2022; Burrell et al. 2021). Understanding the mechanisms that drive these transitions will be critical for managing boreal forest ecosystems in the face of projected climate warming and increasing fire activity.

## Supplementary Information

Supplementary Material 1.

Supplementary Material 2.

## Data Availability

The datasets generated and analyzed in this study are available at the Arctic Data Center, 10.18739/A2BV79X68.

## Abbreviations

AK: Alaska
ALFD: Alaska large fire database
ARH: Adventitious root height
C: Carbon
CWD: Coarse woody debris
CMI: Climate moisture index
DBH: Diameter at breast height
DI: Deciduous index
dNBR: Differenced Normalized Burn Ratio
FRI: Fire return interval
GLMM: Generalized linear mixed-effects models
NECB: Net Ecosystem Carbon Balance
SOL: Soil organic layer
