# Supplementary material for: Increasing wildfire frequency decreases carbon storage and leads to regeneration failure in Alaskan boreal forests
Source: Fire Ecol. 2025 Oct 9;21(1):57. doi: 10.1186/s42408-025-00390-3 (PMC12511247; doi:10.1186/s42408-025-00390-3)
Supplement: Supplementary file 1 — Supplementary Material 1. [file 42408_2025_390_MOESM1_ESM.docx]

Table S1. Number of sites (and residuals) in each pre-fire composition for each fire return interval (FRI). Residuals are from post hoc pairwise comparisons using false discovery rate correction for chi-square test of independence between pre-fire composition class and FRI. Residuals **s**how the direction and magnitude of deviations from expected frequencies, with bolded values representing significance (*p-value<0.05, **^+^**p-value<0.01).

|  | Pre-fire Composition Clas | | | |
| --- | --- | --- | --- | --- |
| Fire Return Interval | Spruce | Mixed | Deciduous | Open |
| LONG | **59 (4.76)*** | 4 (-2.058) | **5 (-3.28)*** | 0 (-1.73) |
| MID | 39 (3.01) | 4 (-0.94) | 3 (-2.70) | 1 (-0.28) |
| SHORT | **10 (-6.71)*** | 10 (2.37) | **23 (6.13)*** | 1 (-0.20) |
| TRIPLE-MID | 6 (-1.47) | 0 (-1.41) | 5 (1.73) | **2 (2.92)^+^** |
| TRIPLE-SHORT | 6 (-1.47) | **5 (2.95)^+^** | 1 (-1.15) | 1 ((1.15) |

Table S2. Pre-fire, post-fire, and fire losses of above- and belowground carbon (C) pools across fire return intervals (FRI; long >70 years, mid >30-70 years, short <30 years, triple-mid: three consecutive fires in <70 years, with the most recent interval of 30-70 years, and triple-short: three consecutive fires in <70 years, with the most recent interval of <30 years). Letters represent significant differences (P < 0.05) among FRIs within the aboveground, belowground, and total (bottom) C pools based on generalized linear mixed models, with site as a random intercept and post-hoc tests with a false discovery rate adjustment for multiple comparisons. Both raw means and model estimated means ± standard error of the mean are presented.

|  | **Pool** | **FRI** | **Modeled mean ± SE** | **Raw Mean ± SE (range)** | **group** |
| --- | --- | --- | --- | --- | --- |
| Prefire | Above | Long | 1316 ± 159 | 1919.15 ± 177.34 (0.00–19051.64) | a |
|  |  | Mid | 1205 ± 176 | 1931.18 ± 279.23 (36.23–25571.10) | a |
|  |  | Short | 1228 ± 186 | 1873.67 ± 194.46 (0.00–13373.06) | a |
|  |  | Triple-Mid | 532 ± 152 | 900.65 ± 192.98 (8.43–5748.01) | b |
|  |  | Triple-Short | 509 ± 145 | 720.20 ± 138.98 (13.66–4280.27) | b |
|  | Below | Long | 7496 ± 418 | 8239.99 ± 274.90 (2453.96–23802.22) | a |
|  |  | Mid | 5469 ± 367 | 6039.07 ± 251.34 (1537.71–17370.60) | b |
|  |  | Short | 4188 ± 291 | 4566.29 ± 211.88 (1556.66–14951.95) | c |
|  |  | Triple-Mid | 5376 ± 686 | 6260.22 ± 606.75 (1656.47–15115.33) | bc |
|  |  | Triple-Short | 5108 ± 652 | 5822.98 ± 526.13 (1690.51–14798.88) | bc |
|  | Total | Long | 9645 ± 472 | 10159.15 ± 277.91 (3161.41–24471.32) | a |
|  |  | Mid | 7199 ± 424 | 7970.25 ± 360.90 (2308.22–30222.06) | b |
|  |  | Short | 6001 ± 365 | 6439.96 ± 264.41 (2436.23–16398.24) | b |
|  |  | Triple-Mid | 6389 ± 715 | 7160.87 ± 617.00 (2385.04–15344.62) | b |
|  |  | Triple-Short | 6073 ± 680 | 6543.18 ± 480.14 (2643.00–14822.74) | b |
| Postfire | Above | Long | 950 ± 129 | 1500.92 ± 155.58 (0.00–18014.57) | a |
|  |  | Mid | 755 ± 124 | 1377.55 ± 231.96 (15.48–21119.85) | a |
|  |  | Short | 709 ± 121 | 1221.45 ± 150.81 (0.00–12157.26) | a |
|  |  | Triple-Mid | 309 ± 98.8 | 543.82 ± 124.22 (0.00–3397.48) | b |
|  |  | Triple-Short | 314 ± 100 | 534.18 ± 128.12 (0.00–4013.17) | b |
|  | Below | Long | 2845 ± 471 | 5082.71 ± 299.62 (0.00–22822.10) | a |
|  |  | Mid | 2049 ± 405 | 3350.17 ± 266.81 (0.00–13512.26) | a |
|  |  | Short | 886 ± 190 | 2041.36 ± 233.33 (0.00–13362.11) | b |
|  |  | Triple-Mid | 2396 ± 898 | 4110.73 ± 657.38 (0.00–14288.84) | ab |
|  |  | Triple-Short | 1573 ± 601 | 3270.13 ± 582.85 (0.00–13212.87) | ab |
|  | Total | Long | 5668 ± 542 | 6583.63 ± 272.66 (253.88–23284.62) | a |
|  |  | Mid | 3752 ± 436 | 4727.72 ± 308.02 (496.11–21119.85) | b |
|  |  | Short | 2148 ± 264 | 3262.81 ± 275.32 (0.00–13790.24) | C |
|  |  | Triple-Mid | 3237 ± 720 | 4654.56 ± 639.25 (156.92–14355.71) | bc |
|  |  | Triple-Short | 2670 ± 598 | 3804.31 ± 542.85 (72.82–13226.20) | bc |
| Loss | Above | Long | 320 ± 36 | 418.22 ± 29.19 (0.00–1796.40) | ab |
|  |  | Mid | 396 ± 53 | 553.63 ± 56.06 (20.75–4451.25) | a |
|  |  | Short | 451 ± 63 | 652.22 ± 64.45 (0.00–3923.93) | a |
|  |  | Triple-Mid | 203 ± 54 | 356.83 ± 83.52 (6.90–2922.42) | bc |
|  |  | Triple-Short | 158 ± 42 | 186.02 ± 27.04 (9.31–749.69) | c |
|  | Below | Long | 2732 ± 214 | 3157.29 ± 143.81 (19.01–11481.26) | a |
|  |  | Mid | 2446 ± 231 | 2688.90 ± 115.28 (0.00–6174.68) | ab |
|  |  | Short | 1889 ± 191 | 2524.93 ± 182.33 (0.00–13477.90) | b |
|  |  | Triple-Mid | 1969 ± 358 | 2149.49 ± 201.42 (0.00–6285.74) | ab |
|  |  | Triple-Short | 2078 ± 379 | 2552.85 ± 310.87 (0.00–8769.76) | ab |
|  | Total | Long | 3145 ± 275 | 3575.52 ± 154.62 (318.54–11817.03) | a |
|  |  | Mid | 2976 ± 313 | 3242.53 ± 146.36 (192.57–9102.21) | a |
|  |  | Short | 2341 ± 254 | 3177.15 ± 191.90 (5.64–13621.17) | a |
|  |  | Triple-Mid | 2307 ± 460 | 2506.32 ± 255.13 (6.90–9208.16) | a |
|  |  | Triple-Short | 2131 ± 426 | 2738.87 ± 320.93 (60.99–8896.53) | a |

Table S3. Pre-fire, post-fire, and fire losses of aboveground carbon (C) pools (trees, shrubs, snags and coarse woody debris), across fire return intervals (FRI; long >70 years, mid >30&70 years, short <30 years, triple-mid: three consecutive fires in <70 years, with the most recent interval of 30-70 years, and triple-short: three consecutive fires in <70 years, with the most recent interval of <30 years). Letters represent significant differences (P < 0.05) among FRIs for each aboveground C pool component based on generalized linear mixed models, with site as a random intercept, and post-hoc tests with a false discovery rate adjustment for multiple comparisons. Both raw means and model estimated means ± standard error of the mean are presented.

|  | **Pool** | **FRI** | **Modeled mean ± SE** | **Raw Mean ± SE (range)** | **group** |
| --- | --- | --- | --- | --- | --- |
| Pre-fire | CWD | Long | 24.8 ± 7.31 | 133.62 ± 31.00 (0.00–3958.83) | a |
|  |  | Mid | 65.7 ± 20.6 | 306.09 ± 62.50 (0.00–4805.61) | bc |
|  |  | Short | 433 ± 122 | 932.40 ± 101.98 (0.00–6097.07) | d |
|  |  | Triple-Mid | 16.6 ± 10.7 | 57.61 ± 31.25 (0.00–1132.20) | ab |
|  |  | Triple-Short | 116.2 ± 62.6 | 203.99 ± 39.34 (0.00–915.93) | c |
|  | Snags | Long | 1.44 ± 1.210 | 55.15 ± 19.29 (0.00–3270.71) | a |
|  |  | Mid | 0.74 ± 0.73 | 27.66 ± 12.77 (0.00–1689.68) | a |
|  |  | Short | 7.77 ± 6.67 | 338.96 ± 96.57 (0.00–7070.29) | a |
|  |  | Triple-Mid | 1.7 ± 2.46 | 78.51 ± 69.34 (0.00–2709.37) | a |
|  |  | Triple-Short | 20.94 ± 23.9 | 140.76 ± 59.34 (0.00–2093.89) | a |
|  | Shrubs | Long | 10.5 ± 3.17 | 55.97 ± 9.87 (0.00–1195.41) | a |
|  |  | Mid | 24.2 ± 8.16 | 104.84 ± 17.91 (0.00–1290.06) | ab |
|  |  | Short | 21.6 ± 7.28 | 53.20 ± 9.47 (0.00–924.29) | ab |
|  |  | Triple-Mid | 121.2 ± 71.2 | 350.87 ± 78.26 (0.00–1858.62) | b |
|  |  | Triple-Short | 36.3 ± 21.6 | 66.52 ± 21.75 (0.00–609.35) | ab |
|  | Trees | Long | 1119 ± 183 | 1680.90 ± 156.08 (0.00–15207.73) | a |
|  |  | Mid | 673 ± 136 | 1492.58 ± 267.71 (0.00–25571.10) | a |
|  |  | Short | 164 ± 36.9 | 549.11 ± 121.99 (0.00–12350.07) | b |
|  |  | Triple-Mid | 152 ± 62.4 | 413.66 ± 125.39 (0.00–3360.20) | b |
|  |  | Triple-Short | 149 ± 60.6 | 308.94 ± 111.98 (0.00–4238.88) | b |
| Posfire | CWD | Long | 9.7 ± 3.06 | 81.20 ± 24.71 (0.00–3071.27) | a |
|  |  | Mid | 26.4 ± 9.0 | 152.95 ± 33.83 (0.00–2574.22) | bc |
|  |  | Short | 187.4 ± 57 | 477.69 ± 61.56 (0.00–3907.83) | d |
|  |  | Triple-Mid | 6.58 ± 4.55 | 27.64 ± 15.16 (0.00–544.88) | ab |
|  |  | Triple-Short | 50.7 ± 29.4 | 97.25 ± 21.18 (0.00–467.46) | cd |
|  | Snags | Long | 1.29 ± 1.05 | 44.59 ± 17.47 (0.00–3143.37) | a |
|  |  | Mid | 0.67 ± 0.64 | 24.31 ± 12.57 (0.00–1689.68) | a |
|  |  | Short | 6.5 ± 5.4 | 246.07 ± 72.57 (0.00–5585.27) | a |
|  |  | Triple-Mid | 0.42 ± 0.64 | 5.86 ± 2.42 (0.00–66.67) | a |
|  |  | Triple-Short | 18.3 ± 20.3 | 131.15 ± 56.96 (0.00–1992.09) | a |
|  | Shrubs | Long | 2.76 ± 1.19 | 26.37 ± 6.03 (0.00–923.55) | a |
|  |  | Mid | 5.35 ± 2.62 | 44.40 ± 9.42 (0.00–665.71) | ab |
|  |  | Short | 4.15 ± 2.12 | 25.51 ± 7.69 (0.00–914.82) | ab |
|  |  | Triple-Mid | 39.21 ± 30.9 | 158.67 ± 49.24 (0.00–1328.77) | b |
|  |  | Triple-Short | 19.39 ± 15.2 | 25.97 ± 7.97 (0.00–267.90) | ab |
|  | Trees | Long | 847 ± 142 | 1353.87 ± 139.92 (0.00–14455.70) | a |
|  |  | Mid | 493 ± 102 | 1155.89 ± 223.60 (0.00–21119.85) | a |
|  |  | Short | 142 ± 32.4 | 472.18 ± 109.51 (0.00–11298.54) | b |
|  |  | Triple-Mid | 121 ± 51.1 | 351.65 ± 112.50 (0.00–2852.60) | b |
|  |  | Triple-Short | 119 ± 49.7 | 279.81 ± 106.21 (0.00–3987.22) | b |
| Loss | CWD | Long | 13.38 ± 3.95 | 52.41 ± 8.79 (0.00–949.82) | a |
|  |  | Mid | 35.47 ± 11.1 | 153.14 ± 30.51 (0.00–2231.40) | bc |
|  |  | Short | 222.64 ± 61.5 | 454.71 ± 49.23 (0.00–2661.25) | d |
|  |  | Triple-Mid | 8.95 ± 5.74 | 29.97 ± 16.23 (0.00–587.32) | ab |
|  |  | Triple-Short | 62.4 ± 33.0 | 106.73 ± 19.73 (0.00–452.02) | c |
|  | Snags | Long | 0 ± 0 | 10.56 ± 5.41 (0.00–779.63) | a |
|  |  | Mid | 0 ± 0 | 3.36 ± 1.21 (0.00–122.01) | a |
|  |  | Short | 0.004 ± 0.01 | 92.89 ± 33.06 (0.00–3210.57) | a |
|  |  | Triple-Mid | 0.01 ± 0.04 | 72.64 ± 69.40 (0.00–2709.37) | a |
|  |  | Triple-Short | 0.02 ± 0.05 | 9.61 ± 5.61 (0.00–195.08) | a |
|  | Shrubs | Long | 4.97 ± 1.58 | 29.60 ± 5.90 (0.00–756.58) | a |
|  |  | Mid | 13.48 ± 4.8 | 60.44 ± 11.87 (0.00–1266.35) | ab |
|  |  | Short | 0.65 ± 2.47 | 27.69 ± 5.44 (0.00–551.64) | a |
|  |  | Triple-Mid | 63.48 ± 40.0 | 192.20 ± 39.25 (0.00–911.34) | b |
|  |  | Triple-Short | 14.56 ± 9.35 | 40.55 ± 16.76 (0.00–531.22) | ab |
|  | Trees | Long | 237.9 ± 41.0 | 327.03 ± 23.27 (0.00–1637.40) | a |
|  |  | Mid | 160 ± 337 | 336.69 ± 47.58 (0.00–4451.25) | a |
|  |  | Short | 13.6 ± 3.37 | 76.92 ± 16.17 (0.00–1051.53) | b |
|  |  | Triple-Mid | 21 ± 9.10 | 62.02 ± 18.09 (0.00–507.61) | b |
|  |  | Triple-Short | 17.2 ± 7.37 | 29.13 ± 7.24 (0.00–251.66) | b |

Table S4. Number of sites (and residuals) in each post-fire composition for each fire return interval (FRI). Residuals are from post hoc pairwise comparisons using false discovery rate correction for chi-square test of independence between post-fire composition classes and FRI in all sites and for the subset of sites that were spruce-dominated pre-fire. Residuals **s**how the direction and magnitude of deviations from expected frequencies, with bolded values representing significance (p-value<0.01).

|  |  | | Post-fire Composition Class | | | | | | | | |  |
| --- | --- | --- | --- | --- | --- | --- | --- | --- | --- | --- | --- | --- |
|  | Fire Return Interval | | | Spruce | | Mixed | | Decid | | Open | | |
| All sites | Long | 26 (2.93) | | | 4 (0.93) | | 6 (-2.14) | | 18 (-1.49) | |  |  |
|  | Mid | 16 (2.08) | | | 3 (1.15) | | 4 (-1.44) | | 10 (-1.32) | |  |  |
|  | Short | **0 (-4.61)** | | | 0 (-1.52) | | **15 (4.32)** | | 17 (1.59) | |  |  |
|  | Triple-Mid | 0 (-2.07) | | | 0 (-0.69) | | 0 (-1.46) | | **8 (3.49)** | |  |  |
|  | Triple-Short | 3 (0.53) | | | 0 (-0.64) | | 2 (0.57) | | 2 (-0.69) | |  |  |
| Spruce sites | Long | 25 (0.87) | | | 4 (0.13) | | 4 (-0.66) | | 13 (-0.58) | |  |  |
|  | Mid | 15 (0.46) | | | 3 (0.56) | | 4 (0.75) | | 6 (-1.34) | |  |  |
|  | Short | 0 (-2.54) | | | 0 (-0.77) | | 1 (0.49) | | 5 (2.88) | |  |  |
|  | Triple-Mid | 0 (-1.01) | | | 0 (-0.30) | | 0 (-0.35) | | 1 (1.50) | |  |  |
|  | Triple-Short | 2 (0.58) | | | 0 (-0.53) | | 0 (-0.61) | | 1 (0.09) | |  |  |

Table S5. Deciduous and conifer seeding density for all sites and the pre-fire spruce dominated sites across fire return intervals (FRI; long >70 years, mid >30&70 years, short <30 years, triple-mid: three consecutive fires in <70 years, with the most recent interval of 30-70 years, and triple-short: three consecutive fires in <70 years, with the most recent interval of <30 years). Letters represent significant differences (P < 0.05) among FRIs based on generalized linear models and post-hoc tests with a false discovery rate adjustment for multiple comparisons. Both raw means and model estimated means ± standard error of the mean are presented.

|  |  | **Spruce Density** | | | **Deciduous Density** | | |
| --- | --- | --- | --- | --- | --- | --- | --- |
|  | **FRI** | **Modeled mean ± SE** | **Raw Mean ± SE (range)** | **group** | **Modeled mean ± SE** | **Raw Mean ± SE (range)** | **group** |
| All sites | Long | 2.4 ± 0.6 | 2.4 ± 0.8 (0-36) | ab | 0.5 ± 0.2 | 0.5 ± 0.2 (0-11.4) | a |
|  | Mid | 4.7 ± 1.3 | 4.7 ± 1.3 (0-32.9) | b | 3.2 ± 1.0 | 3.2 ± 1.7 (0-54.9) | b |
|  | Short | 0.03 ± 0.02 | 0.03 ± 0.03 (0-0.7) | c | 0.9 ± 0.4 | 0.9 ± 0.3 (0-8.4) | a |
|  | Triple-Mid | - | 0 | - | - | 0 |  |
|  | Triple-Short | 0.2 ± 0.3 | 0.2 ± 0.1 (0-0.7) | ac | 0.6 ± 0.6 | 0.6 ± 0.4 (0-2.8) | ab |
| Spruce sites | Long | 2.7 ± 0.7 | 2.7 ± 0.9 (0-36) | a | 0.6 ± 0.2 | 0.6 ± 0.3 (0-11.4) | a |
|  | Mid | 5.4 ± 1.6 | 5.4 ± 1.5 (0-32.9) | a | 3.8 ± 1.2 | 3.8 ± 2.0 (0-54.9) | b |
|  | Short | 0.1 ± 0.1 | 0.1 ± 0.1 (0-0.7) | b | 0.1 ± 0.1 | 0.1 ± 0.1 (0-0.4) | a |
|  | Triple-Mid | - | 0 |  | - | 0 | - |
|  | Triple-Short | 0.4 ± 0.6 | 0.4 ± 0.2 (0-0.7) | ab | - | 0 |  |


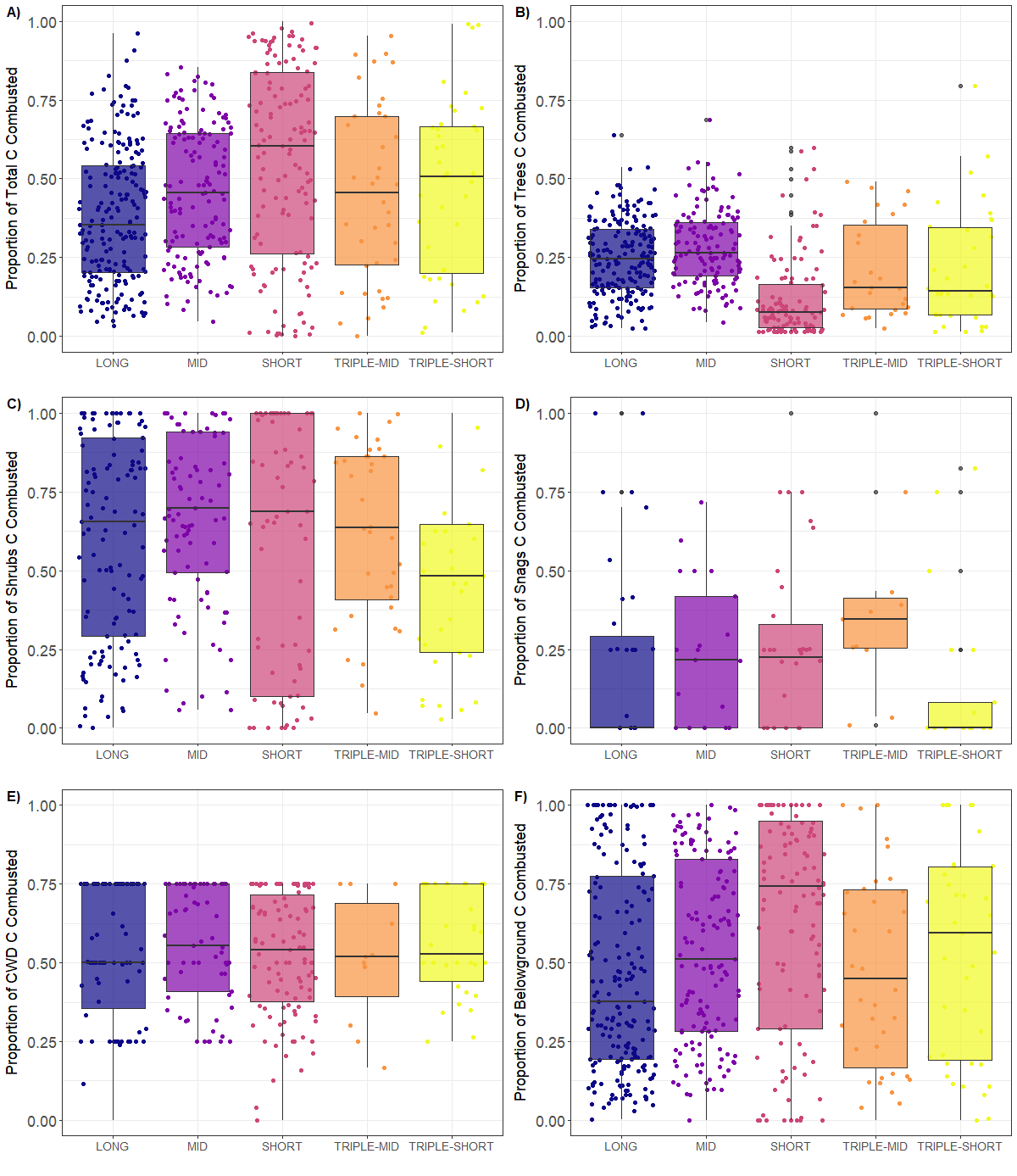


Figure S1. Proportion of A) Total, B) Trees, C) Shrubs, D) Snags, E) Coarse woody debris (CWD), and F) Belowground pre-fire carbon pools combusted among fire return intervals.


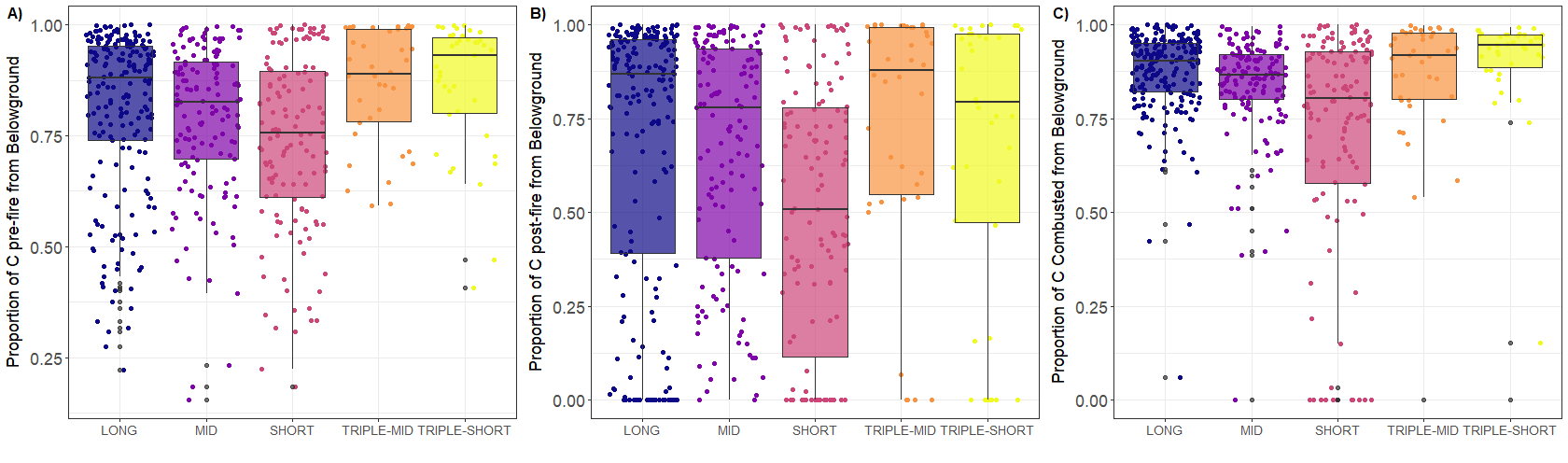


Figure S2. The proportion of total carbon (C) from belowground in A) pre-fire and B) post-fire stands, and C) combusted from wildfire across different fire return intervals.


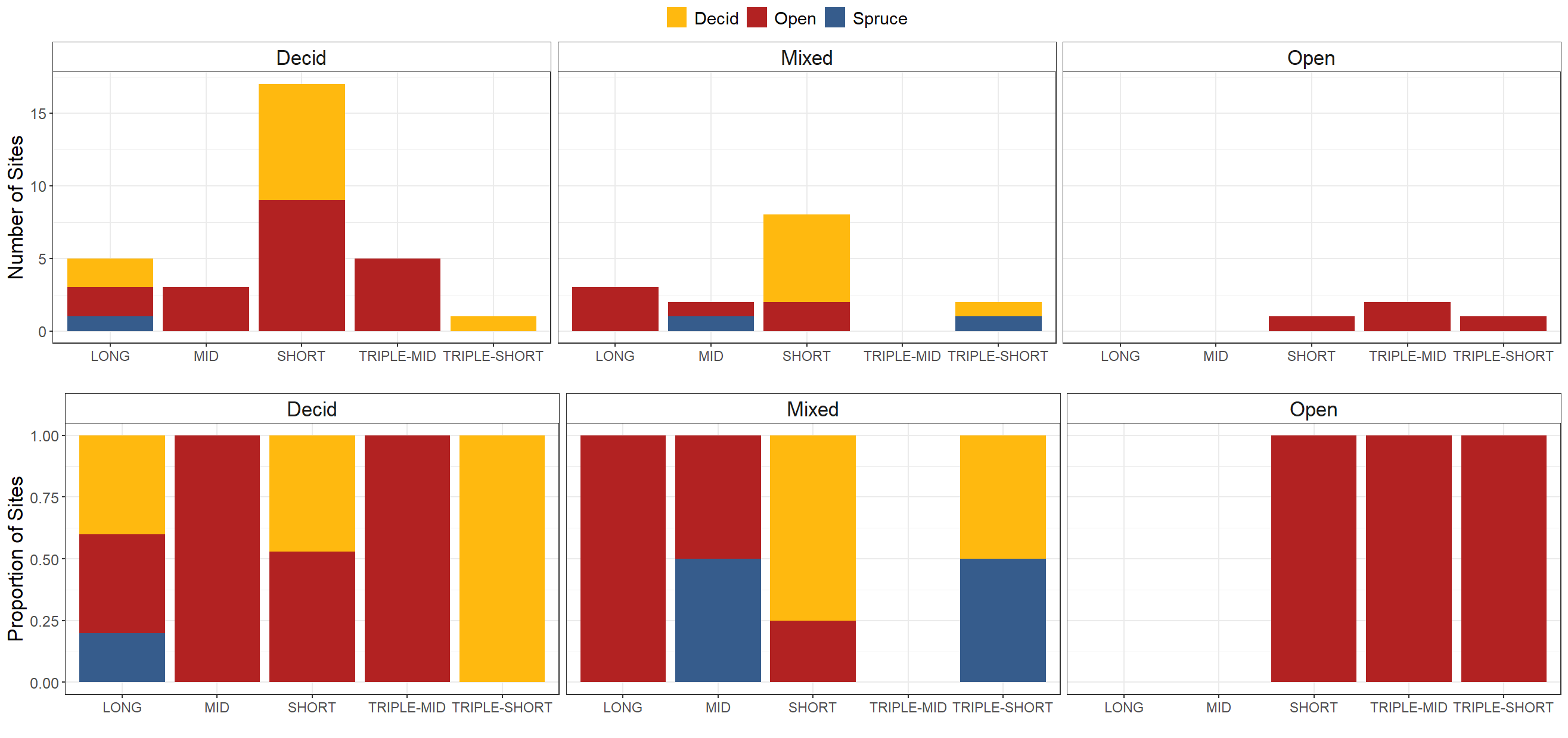


Figure S3. Number (top panel) and proportion (bottom panel) of pre-fire deciduous, mixed, or open sites in each fire return interval that exhibited post-fire regeneration failure (red), deciduous dominance (yellow), or spruce dominance (blue).
