# Supplementary material for: Increasing wildfire frequency decreases carbon storage and leads to regeneration failure in Alaskan boreal forests
Source: Fire Ecol. 2025 Oct 9;21(1):57. doi: 10.1186/s42408-025-00390-3 (PMC12511247; doi:10.1186/s42408-025-00390-3)
Supplement: Supplementary file 2 — Supplementary Material 2. [file 42408_2025_390_MOESM2_ESM.docx]

**Supplementary material 2: Pre-fire soil organic layer (SOL) predictions**

To estimate pre-fire SOL depth, we used Alaska forest inventory data from our study ecoregions. We acquired data from forest inventory databases: the US Forest Service Alaska Forest Inventory Analysis (Burrill et al. 2021), the Cooperative Alaska Forest Inventory (Malone et al. 2009), and the Bonanza Creek LTER Regional Site Network (Ruess 2015, Van Cleve et al. 2021, Ruess et al. 2023); and from studies that included a forest inventory (Alexander and Bonanza Creek LTER 2014, Melvin and Bonanza Creek LTER 2018, Mack et al. 2021, Jean et al. 2022, Melvin et al. 2022). Additionally we included SOL depth measurements from plots from this study that we were able to acquire black spruce adventitious root heights for and thus were able to successfully model pre-fire SOL depths using standard SOL depth projection methods (Boby et al. 2010). The data we compiled included SOL measurements as well as stand and landscape characteristics from unburned stands (Table 1). In a few cases, topographic metrics were not available for a subset of the data and so we extracted some elevations and slopes from digital elevation maps using the terra package in R (Hijmans 2024). Additionally, we used the ClimateNA R package (Wang et al. 2016, Burnett 2024) to extract annual climate normal (30-year mean) metrics downscaled to a 30 m DEM of Alaska (U. S. Geological Survey 2021) resampled to 90 m resolution to speed up processing time (Table 1).

For each stand type (conifer, mixed, aspen, birch, and sites with no trees), we trained random forest models to predict pre-fire soil depth. The only plots classified as without trees were ones where all three plots in a site contained no trees. Otherwise, adjacent plot species compositions were averaged to infer the stand type of tree-less plots. Training data for tree-less sites included all available sample data with stand age less than 75 years and biomass less than 3000 g m^-2^. We examined many possible predictor variables including stand biomass and density, slope, aspect, elevation, stand age, soil moisture class, and several different climate normal metrics extracted from ClimateNA. We refined our predictor variable lists by getting rid correlated variables and selecting the top predictors of soil depth using random forest generated variable importance plots. Ultimately for each stand type we tested all combinations of the variables: density, elevation, slope, stand age, and the top two non-correlated climate metrics for each stand type. We required final trained models to retain density as one of the predictors as it gave us intra-site variation. We assessed the strength of all predictor combination-candidate random forest models by comparing model adjusted R^2^, Root Mean Squared Error (RMSE), and Mean Absolute Error (MAE). We also assessed the ability of each model to accurately predict soil depth (based on predicted versus actual depth R^2^ adj.) when the inventory data was split into training (80%) and validation (20%) data. We opted for models that minimized error while maximizing fit between predicted and actual soil depth on split data frames (Table 2). The final models used a repeated cross-validation resampling method, 10 k-folds, three repeats, 1,000 trees, and model RMSE as a metric for selecting the optimal model. We also used grid searches to select the optimal mtry (number of predictors randomly selected at each tree split) for each model (Table 2). Model performance ranged from R^2^ = 0.54 to 0.83 and RMSE ranged from 1.6 cm to 3.8 cm (Table 2). The random forest models were then used to predict pre-fire soil depth in our sample data. The models were created using the R packages randomForest (Cutler and Wiener 2022) and caret (Kuhn et al. 2023).

**Table 1: Data Used to Train Soil Depth Models**

**.** Attributes of data used to train unburned soil depth models (Training Data) compared with our data (Sample Data). Means, standard errors, and range (minimum and maximum) of each variable assessed for inclusion in models are reported. Training data variables in bold were used as predictors in the final soil depth prediction models for corresponding stand type. All climatic variables are 30-mean normals for 1980-2010 extracted using ClimateNA (Wang et al. 2016, Burnett 2024).

|  |  | **Training Data** | | | | **Sample Data** | | | |
| --- | --- | --- | --- | --- | --- | --- | --- | --- | --- |
| **Variable** | **Stand** | **Mean** | **SE** | **Min** | **Max** | **Mean** | **SE** | **Min** | **Max** |
| SOL Depth (cm) | Conifer | 10.9 | 0.2 | 0 | 78.4 |  |  |  |  |
|  | Mixed | 7.5 | 0.3 | 0 | 29.5 |  |  |  |  |
|  | Aspen | 4.2 | 0.2 | 0 | 17.1 |  |  |  |  |
|  | Birch | 6.7 | 0.2 | 0.5 | 36.7 |  |  |  |  |
|  | No Trees | 9.5 | 0.2 | 0 | 45.5 |  |  |  |  |
| Latitude (N) | Conifer | 64.15 | 0.03 | 61.33 | 66.93 | 65.59 | 0.04 | 63.83 | 66.92 |
|  | Mixed | 64.45 | 0.06 | 61.69 | 67.03 | 65.98 | 0.2 | 63.84 | 66.88 |
|  | Aspen | 64.7 | 0.07 | 61.81 | 66.37 | 65.98 | 0.12 | 64.72 | 66.73 |
|  | Birch | 64.76 | 0.04 | 61.91 | 67.03 | 65.71 | 0.11 | 63.83 | 66.86 |
|  | No Trees | 64.71 | 0.03 | 61.38 | 66.88 | 65.58 | 0.08 | 64.48 | 66.86 |
| Longitude (W) | Conifer | 146.84 | 0.07 | 153.78 | 141.01 | 148.65 | 0.15 | 152.78 | 142.77 |
|  | Mixed | 147.71 | 0.17 | 153.79 | 141.03 | 146.31 | 0.75 | 152.78 | 142.78 |
|  | Aspen | 147.6 | 0.16 | 151.94 | 141.19 | 145.43 | 0.52 | 153.79 | 142.77 |
|  | Birch | 148.19 | 0.11 | 153.75 | 141.02 | 147.66 | 0.53 | 153.76 | 142.77 |
|  | No Trees | 147.49 | 0.1 | 153.79 | 141.01 | 148.43 | 0.39 | 153.78 | 142.77 |
| Elevation (m) | Conifer | 1387 | 23 | 77 | 4100 | 343 | 10 | 76 | 852 |
|  | Mixed | 746 | 43 | 102 | 2900 | 299 | 28 | 102 | 539 |
|  | Aspen | 605 | 34 | 125 | 3100 | 321 | 27 | 138 | 528 |
|  | Birch | 659 | 30 | 102 | 2700 | 284 | 13 | 102 | 528 |
|  | No Trees | 839 | 26 | 77 | 3700 | 330 | 20 | 76 | 676 |
| Slope (°) | Conifer | 5.6 | 0.1 | 0 | 43.5 | 5.7 | 0.3 | 0 | 34 |
|  | Mixed | 7.6 | 0.4 | 0 | 34 | 4.7 | 1.4 | 0 | 19 |
|  | Aspen | 6.3 | 0.5 | 0 | 36.9 | 6 | 1.1 | 0 | 19 |
|  | Birch | 9.8 | 0.3 | 0 | 38.7 | 4.8 | 0.7 | 0 | 21 |
|  | No Trees | 5.6 | 0.2 | 0 | 34 | 3.1 | 0.3 | 0 | 10 |
| Moisture Class | Conifer | 2.2 | 0 | 1 | 3 | 2.1 | 0 | 1 | 3 |
|  | Mixed | 1.9 | 0 | 1 | 3 | 1.9 | 0.2 | 1 | 3 |
|  | Aspen | 2.1 | 0 | 1 | 3 | 1.2 | 0.1 | 1 | 3 |
|  | Birch | 1.9 | 0 | 1 | 3 | 1.7 | 0.1 | 1 | 3 |
|  | No Trees | 2.1 | 0 | 1 | 3 | 1.7 | 0.1 | 1 | 3 |

|  |  | **Training Data** | | | | **Sample Data** | | | |
| --- | --- | --- | --- | --- | --- | --- | --- | --- | --- |
| **Variable** | **Stand** | **Mean** | **SE** | **Min** | **Max** | **Mean** | **SE** | **Min** | **Max** |
| Stand Age (yrs) | Conifer | 82 | 1 | 0 | 255 | 72 | 2 | 11 | 255 |
|  | Mixed | 62 | 2 | 15 | 196 | 56 | 8 | 11 | 124 |
|  | Aspen | 36 | 2 | 6 | 120 | 46 | 7 | 11 | 103 |
|  | Birch | 43 | 1 | 6 | 175 | 29 | 3 | 11 | 123 |
|  | No Trees | 33 | 1 | 0 | 74 | 24 | 1 | 15 | 53 |
| Biomass (g m^2^) | Conifer | 3917 | 107 | 0 | 52935 | 3508 | 420 | 0 | 65377 |
|  | Mixed | 7102 | 335 | 46 | 26574 | 8078 | 2022 | 0 | 30133 |
|  | Aspen | 4360 | 298 | 6 | 22632 | 1586 | 322 | 0 | 5585 |
|  | Birch | 6330 | 273 | 0 | 26658 | 2217 | 662 | 0 | 31204 |
|  | No Trees | 835 | 27 | 0 | 2996 | 0 | 0 | 0 | 0 |
| Density (n/m) | Conifer | 0.97 | 0.07 | 0 | 30 | 0.26 | 0.01 | 0 | 1.45 |
|  | Mixed | 1.46 | 0.18 | 0.01 | 21 | 0.35 | 0.06 | 0 | 1 |
|  | Aspen | 1.53 | 0.28 | 0.01 | 27 | 0.16 | 0.02 | 0 | 0.55 |
|  | Birch | 1.44 | 0.21 | 0 | 28 | 0.17 | 0.03 | 0 | 1.1 |
|  | No Trees | 1.65 | 0.14 | 0 | 30 | 0 | 0 | 0 | 0 |
| Annual Heat Moisture Index | Conifer | 19.4 | 0.1 | 5.6 | 33.5 | 18 | 0.2 | 8.7 | 25.8 |
|  | Mixed | 21.1 | 0.2 | 8.2 | 31.8 | 17.3 | 0.7 | 10.4 | 23.1 |
|  | Aspen | 20.1 | 0.2 | 14.3 | 34.5 | 17 | 0.4 | 15.9 | 24.3 |
|  | Birch | 20.7 | 0.2 | 8 | 30.3 | 20 | 0.7 | 10.4 | 28.6 |
|  | No Trees | 19.7 | 0.1 | 8.8 | 31.1 | 18.9 | 0.5 | 10 | 28.6 |
| Climate Moisture Deficit (mm) | Conifer | 140 | 1 | 0 | 296 | 144 | 2 | 64 | 228 |
|  | Mixed | 152 | 2 | 40 | 300 | 158 | 10 | 97 | 227 |
|  | Aspen | 167 | 4 | 40 | 301 | 158 | 10 | 90 | 221 |
|  | Birch | 142 | 2 | 38 | 239 | 175 | 7 | 90 | 277 |
|  | No Trees | 150 | 2 | 17 | 281 | 159 | 5 | 86 | 278 |
| Climate Moisture Index (mm) | Conifer | 5.71 | 0.26 | -13.19 | 78.43 | 4.57 | 0.47 | -8.32 | 23.58 |
|  | Mixed | 3.49 | 0.65 | -12.65 | 78 | 1.9 | 1.64 | -7.69 | 13.79 |
|  | Aspen | 0.12 | 0.56 | -12.79 | 21.14 | 2.36 | 1.52 | -6.97 | 14.31 |
|  | Birch | 5.76 | 0.59 | -13.71 | 81.15 | -0.4 | 1.17 | -15.97 | 18.82 |
|  | No Trees | 3.42 | 0.32 | -13.71 | 67.81 | 1.69 | 1.03 | -15.97 | 23.33 |
| Days above 5 °C | Conifer | 912 | 4 | 343 | 1226 | 976 | 5 | 761 | 1148 |
|  | Mixed | 999 | 7 | 585 | 1213 | 949 | 22 | 784 | 1148 |
|  | Aspen | 988 | 5 | 605 | 1200 | 922 | 19 | 784 | 1160 |
|  | Birch | 1012 | 6 | 455 | 1235 | 1036 | 18 | 784 | 1233 |
|  | No Trees | 961 | 4 | 455 | 1235 | 1008 | 13 | 759 | 1233 |
| Mean Annual Precip. (mm) | Conifer | 379 | 2 | 217 | 995 | 358 | 4 | 218 | 528 |
|  | Mixed | 367 | 6 | 237 | 1020 | 322 | 16 | 223 | 425 |
|  | Aspen | 340 | 5 | 224 | 496 | 322 | 15 | 223 | 440 |
|  | Birch | 385 | 5 | 234 | 1054 | 319 | 9 | 215 | 468 |
|  | No Trees | 361 | 3 | 217 | 916 | 344 | 8 | 215 | 506 |
|  |  | **Training Data** | | | | **Sample Data** | | | |
| **Variable** | **Stand** | **Mean** | **SE** | **Min** | **Max** | **Mean** | **SE** | **Min** | **Max** |
| Mean Annual Temp. (°C) | Conifer | -2.94 | 0.03 | -6.2 | 0.3 | -3.65 | 0.08 | -6.4 | -1.1 |
|  | Mixed | -2.49 | 0.06 | -6.2 | 1.4 | -4.47 | 0.34 | -6.5 | -1.8 |
|  | Aspen | -3.26 | 0.09 | -5.7 | -0.7 | -4.51 | 0.3 | -6.5 | -1.8 |
|  | Birch | -2.3 | 0.05 | -6.2 | 1.4 | -3.83 | 0.17 | -6 | -1.7 |
|  | No Trees | -3.07 | 0.04 | -6.2 | -0.3 | -3.75 | 0.12 | -6.2 | -1.8 |
| Precip. as Snow (mm) | Conifer | 135 | 1 | 56 | 609 | 127 | 2 | 95 | 236 |
|  | Mixed | 123 | 3 | 65 | 464 | 119 | 5 | 98 | 159 |
|  | Aspen | 115 | 1 | 61 | 213 | 125 | 5 | 98 | 164 |
|  | Birch | 128 | 3 | 61 | 489 | 119 | 3 | 99 | 207 |
|  | No Trees | 125 | 1 | 58 | 442 | 127 | 4 | 99 | 234 |
| Relative Humidity (%) | Conifer | 59 | 0.1 | 53 | 67 | 60.8 | 0.2 | 56 | 67 |
|  | Mixed | 59.5 | 0.2 | 54 | 67 | 60.4 | 0.5 | 56 | 66 |
|  | Aspen | 58.5 | 0.2 | 54 | 66 | 60.4 | 0.3 | 59 | 64 |
|  | Birch | 60.6 | 0.1 | 52 | 67 | 60.8 | 0.3 | 56 | 66 |
|  | No Trees | 59.6 | 0.1 | 54 | 67 | 59.9 | 0.2 | 56 | 64 |
| Continentality (°C) | Conifer | 35 | 0 | 18 | 46 | 38 | 0 | 32 | 46 |
|  | Mixed | 36 | 0 | 24 | 46 | 40 | 1 | 32 | 46 |
|  | Aspen | 37 | 0 | 25 | 44 | 40 | 1 | 32 | 46 |
|  | Birch | 35 | 0 | 24 | 42 | 39 | 0 | 32 | 46 |
|  | No Trees | 36 | 0 | 26 | 46 | 38 | 0 | 36 | 46 |

Table 2: Soil Depth Random Forest Model Fit Statistics

. Model fit statistics (R^2^, RMSE, and MAE, final mtry) and the % increase in Mean Squared Error (MSE) that each variable’s removal would cause are reported. Additionally, R^2^ adjusted values of the linear relationships between predicted and actual soil depth when we trained the data models on a random subset of the data and tested it on the remainder (80% train and 20% test data). The linear relationships between predicted and actual soil depth were significant (P < 0.05) for all of the split data models. NA under any given variable’s %increase in MSE means it wasn’t used the final model to predict soil depths for that stand type.

| **Forest Type** | **Conifer** | **Mixed** | **Aspen** | **Birch** | **No Trees** |
| --- | --- | --- | --- | --- | --- |
| N of Training Data | 1907 | 294 | 290 | 446 | 1085 |
| mtry | 2 | 2 | 3 | 3 | 4 |
| Model R^2^ | 0.82 | 0.55 | 0.66 | 0.46 | 0.79 |
| Model RMSE | 3.8 | 3.1 | 1.6 | 3.0 | 3.4 |
| Model MAE | 2.7 | 2.1 | 1.1 | 2.0 | 2.3 |
| Variable % Increase MSE |  |  |  |  |  |
| Elevation | 104.8 | 17.4 | 7.5 | 15.2 | 93.7 |
| Density | 10.8 | 5.5 | 2.2 | 1.8 | 12.6 |
| Stand Age | 30.9 | 9.0 | 5.5 | 7.1 | 46.6 |
| Slope | NA | NA | 1.8 | 2.7 | NA |
| AHM | 25.0 | 9.9 | NA | 9.9 | NA |
| CMD | NA | NA | NA | NA | 41.0 |
| DD5 | NA | NA | 6.7 | NA | NA |
| RH | 25.9 | NA | NA | NA | 18.3 |
| TD | NA | 6.9 | NA | NA | NA |
| Train/Test R2 adj. | 0.83 | 0.69 | 0.58 | 0.54 | 0.78 |

**References:**

Alexander, H. D. and Bonanza Creek LTER. 2014. Size and composition of all live and dead trees and large shrubs across a compositional gradient of intermediate-aged and mature forest stands within Interior Alaska collected 2008-2011. Environmental Data Initiative.

Alexander, H. D., and M. C. Mack. 2016. A Canopy Shift in Interior Alaskan Boreal Forests: Consequences for Above- and Belowground Carbon and Nitrogen Pools during Post-fire Succession. Ecosystems 19:98–114.

Boby, L. A., E. A. G. Schuur, M. C. Mack, D. L. Verbyla, and J. F. Johnstone. 2010. Quantifying fire severity, carbon, and nitrogen emissions in Alaska’s boreal forest. Ecological Applications 20:1633–1647.

Burnett, M. 2024, November 28. burnett-m/climatenaR. R.

Burrill, E. A., A. M. DiTommaso, J. A. Turner, Scott. A. Pugh, G. Christensen, C. J. Perry, and B. L. Conkling. 2021. The Forest Inventory and Analysis Database: Database Description and User Guide for Phase 2 (version 9.0.1). Page 1026.

Cutler, F. original by L. B. and A., and R. port by A. L. and M. Wiener. 2022, May 23. randomForest: Breiman and Cutler’s Random Forests for Classification and Regression.

Hijmans, R. J. 2024. terra: Spatial Data Analysis.

Jean, M., H. D. Alexander, M. C. Mack, J. F. Johnstone, and Bonanza Creek LTER. 2022. Site Location and Environmental Characteristics for 83 Locations of 6-163 Years Old Black Spruce, Alaska Paper Birch, and Aspen Stands Across Interior Alaska. Sampled in 2008-2010 and 2013-2015. Environmental Data Initiative.

Kuhn, M., J. Wing, S. Weston, A. Williams, C. Keefer, A. Engelhardt, T. Cooper, Z. Mayer, B. Kenkel, R Core Team, M. Benesty, R. Lescarbeau, A. Ziem, L. Scrucca, Y. Tang, C. Candan, and T. Hunt. 2023, March 21. caret: Classification and Regression Training.

Mack, M. C., X. J. Walker, J. F. Johnstone, H. D. Alexander, A. M. Melvin, M. Jean, S. N. Miller, and Bonanza Creek LTER. 2021. A Chronosequence of Biomass and Carbon and Nitrogen Stocks Across Boreal Deciduous, Mixed, and Black Spruce Forests in Interior Alaska. Environmental Data Initiative.

Malone, T., J. Liang, and E. C. Packee. 2009. Cooperative Alaska Forest Inventory. Page PNW-GTR-785. U.S. Department of Agriculture, Forest Service, Pacific Northwest Research Station, Portland, OR.

Melvin, A. M. and Bonanza Creek LTER. 2018. Soil characteristics and nutrient pools and fluxes for Murphy Dome study site. Environmental Data Initiative.

Melvin, A. M., M. C. Mack, and Bonanza Creek LTER. 2022. Tree inventory for adjacent stands of Picea mariana and Betula neoalaskana located in the 1958 Murphy Dome fire scar - 2012. Environmental Data Initiative.

Ruess, R. W. 2015. Organic Horizon Depth in the Regional Site Network, Bonanza Creek LTER. Bonanza Creek LTER, University of Alaska Fairbanks.

Ruess, R. W., M. C. Mack, and J. Hollingsworth. 2023. Bonanza Creek LTER Regional Site Network Site Information Collected 2013-2015. Bonanza Creek LTER, University of Alaska Fairbanks.

U. S. Geological Survey. 2021. USGS 1 Arc Second Digital Elevation Model. The National Map.

Van Cleve, K., F. S. Chapin III, R. Ruess, and Bonanza Creek LTER. 2021. Bonanza Creek LTER: Tree Inventory Data from 1989 to present at Core research sites in Interior Alaska. Bonanza Creek LTER, University of Alaska Fairbanks.

Wang, T., A. Hamann, D. L. Spittlehouse, and C. Carroll. 2016. Locally Downscaled and Spatially Customizable Climate Data for Historical and Future Periods for North America. PLOS ONE 11:e0156720.
